# Reproductive Systems in *Paspalum*: Relevance for Germplasm Collection and Conservation, Breeding Techniques, and Adoption of Released Cultivars

**DOI:** 10.3389/fpls.2019.01377

**Published:** 2019-11-21

**Authors:** Carlos A. Acuña, Eric J. Martínez, Alex L. Zilli, Elsa A. Brugnoli, Francisco Espinoza, Florencia Marcón, Mario H. Urbani, Camilo L. Quarin

**Affiliations:** Instituto de Botánica del Nordeste, Consejo Nacional de Investigaciones Científicas y Técnicas, Facultad de Ciencias Agrarias, Universidad Nacional del Nordeste, Corrientes, Argentina

**Keywords:** apomixis, polyploidy, plant collection, germplasm conservation, breeding methods

## Abstract

The objective of this review is to analyze and describe the impact that mode of reproduction in *Paspalum* has on germplasm conservation, genetic improvement, and commercialization of cultivars. Germplasm collection and conservation can now be rethought considering the newly available information related to how diversity is allocated in nature and how it can be transferred between the sexual and apomictic germplasm using novel breeding approaches. An inventory of species and accessions conserved around the world is analyzed in relation to the main germplasm banks. Because of the importance of apomixis in *Paspalum* species different breeding approaches have been used and tested. Knowledge related to the inheritance of apomixis, variable expressivity of the trait and techniques for early identification of apomicts has helped to improve the efficiency of the breeding methods. Novel breeding techniques are also being developed and are described regarding its advantages and limitations. Finally, the impact of reproductive mode on the adoption of the released cultivars is discussed.

## Introduction

*Paspalum* L. is a large genus of the Poaceae with nearly 310 species that are mainly distributed in the Americas (Morrone et al., 2012). *Paspalum* species are well represented in rangelands used for cattle production systems, but some species are also cultivated for forage, turf, and cereal. Although modes of reproduction are highly variable within *Paspalum*, polyploidy and apomixis are common, affecting allocation of plant diversity among species and populations. The richness of information available for the genus allowed us to review the impact of mode of reproduction on germplasm collection and conservation, genetic improvement, and adoption of the released cultivars.

## Population Diversity And Germplasm Collection

**Figure 1:**
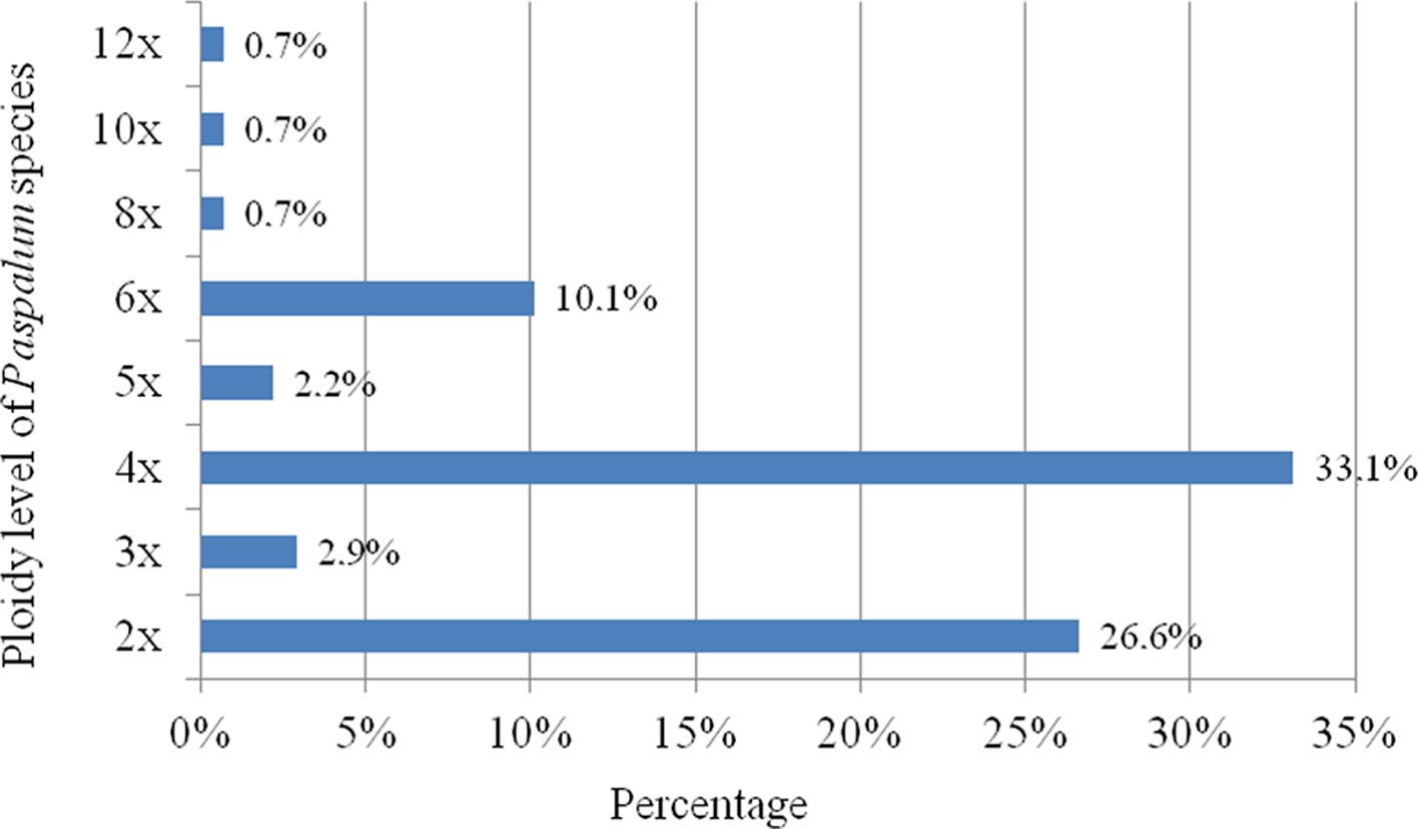
Proportion of species for each ploidy level in *Paspalum* genus (according to Ortiz et al., 2013).

Ploidy level and mode of reproduction have a great impact on species genetic diversity. Germplasm collection and conservation of the genetic variation contained within species are the basis for selection as well as for plant improvement. *Paspalum* is one of the largest genera of the Panicoideae subfamily with the great majority of its species being native to the Americas, distributed throughout the tropics, subtropics, and temperate regions (Zuloaga and Morrone, 2005; Rua et al., 2010; Morrone et al., 2012). Ploidy levels in *Paspalum* are variable, ranging from 2*x* to 12*x*. The tetraploid and diploid cytotypes are most common in nature (Figure 1). Mode of reproduction of 72 *Paspalum* species has been determined; 22 reproduce exclusively by apomixis, 27 by sexuality and 23 reproduce by both reproductive paths, but at different ploidy levels (Ortiz et al., 2013).

For many *Paspalum* species, there are several individual collections and chromosomal records, while there are still a few species whose genetic system and population diversity have been characterized. In the past two decades, extensive collections of germplasm and population diversity analysis have been made for some *Paspalum* species (Urbani et al., 2002; Daurelio et al., 2004; Cidade et al., 2008; Sartor et al., 2011; Brugnoli et al., 2013; Upadhyaya et al., 2014; Eudy et al., 2017). The patterns of genetic diversity in these natural populations have been determined using morphological, ecological, cytological, and molecular traits. Most of these species form agamic complexes with sexual diploid and apomictic polyploid cytotypes, e.g., *P. notatum* Flüggé (Gates et al., 2004), *P. simplex* Morong ex Britton (Caponio and Quarin, 1987; Espinoza and Quarin, 1997), *Paspalum alcalinum* Mez, syn. *P. buckleyanum* Vasey (Burson, 1997; Sartor et al., 2011), *P. denticulatum* Trin. (Quarin and Burson, 1991; Sartor et al., 2011) and *P. rufum* Nees (Burson, 1975; Norrmann et al., 1989; Sartor et al., 2011; Sartor et al., 2013). *Paspalum dilatatum* Poir and *P. scrobiculatum* L. are two examples of agamic complexes, but at the polyploid level (Bashaw et al., 1970; Pritchard, 1970; Chao, 1974; Quarin and Hanna, 1980; Evers and Burson, 2004). The available information related to population diversity, ploidy levels and mode of reproduction for some of the *Paspalum* species can be used to devise strategies for germplasm collection and conservation.

### *Paspalum notatum* and *P. simplex*

*Paspalum notatum* is an important component of rangelands in South America, and is used as cultivated pasture and turf in warm, humid areas (Blount and Acuña, 2009). It is a multiploid species with sexual and cross-pollinated diploid and apomictic tetraploid cytotypes (Burton, 1948; Burton, 1955). Some triploids and pentaploids have been reported (Quarin, 1992; Tischler and Burson, 1995; Daurelio et al., 2004). The tetraploid cytotype is naturally distributed throughout the range of the species, from northern Mexico to central Argentina, while the diploid is restricted to Northeastern Argentina (Gates et al., 2004). Daurelio et al. (2004) analyzed the genetic structure of three natural populations of *P. notatum* using molecular markers; one was diploid, another was tetraploid, sympatric to the diploid, and the third was tetraploid and allopatric. Greater variability was observed within the tetraploid population sympatric to the diploid, indicating that sympatry of diploid and tetraploid populations seemed to promote the generation of variability in apomictic systems by interploidy gene flow, as it was previously stated by Quarin (1992).

**Figure 2:**
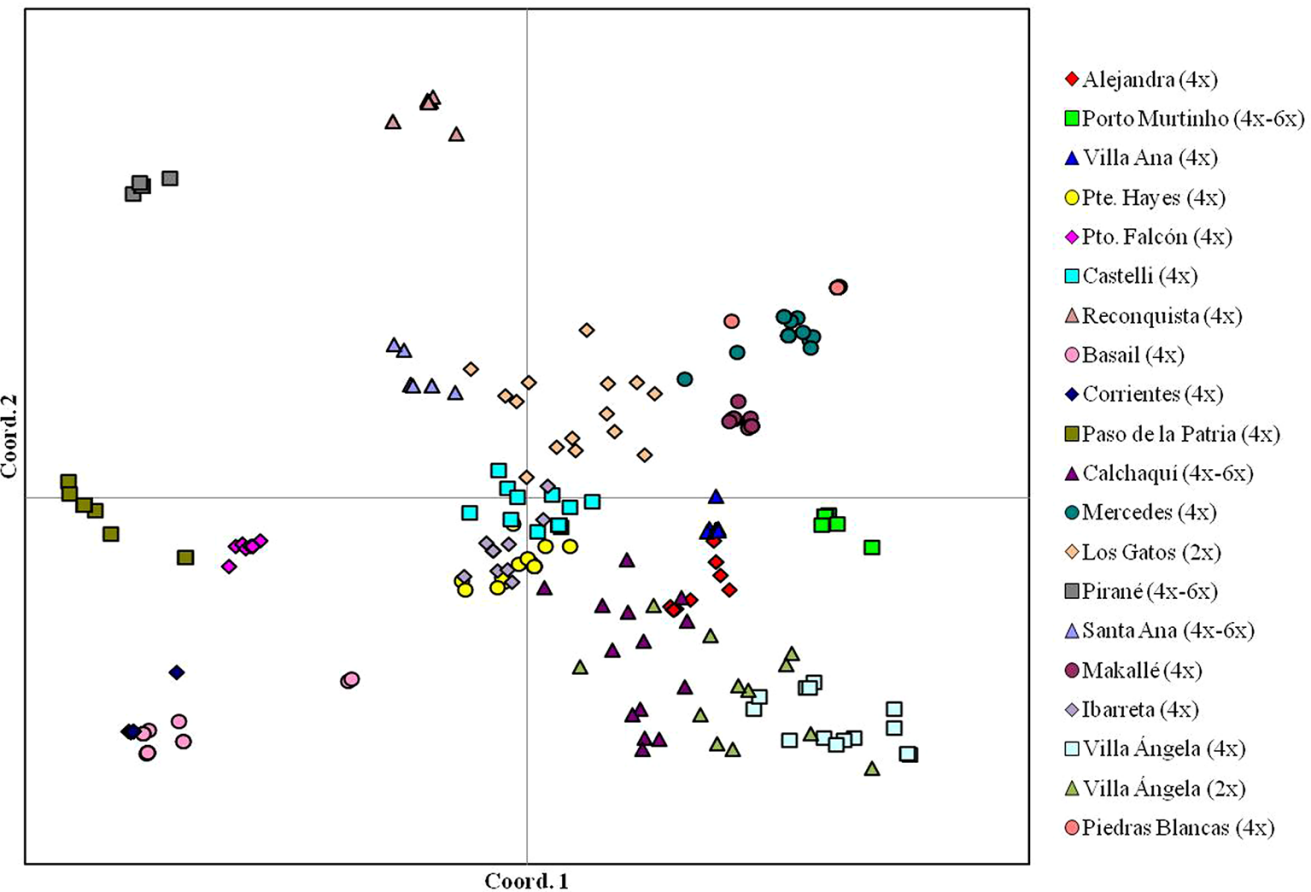
Intra-population diversity in 20 natural populations of *Paspalum simplex* based on inter-simple sequence repeat molecular marker data (adapted from Brugnoli et al., 2013 and Brugnoli et al., 2014).

*Paspalum simplex* is a species native to South America (Urbani et al., 2002) with forage potential for semi-arid regions (Brugnoli et al., 2014). Diploid, triploid, tetraploid, and hexaploid genotypes were reported, the tetraploid being the most frequent (Urbani et al., 2002). A diversity analysis of diploid, tetraploid, and mixed diploid-tetraploid populations of *P. simplex* showed a greater genetic variation in the tetraploid populations sympatric to a diploid population than tetraploid allopatric populations (Brugnoli et al., 2013). Tetraploid allopatric populations are more uniform and generally a single genotype predominates. A Principal Coordinates Analysis (PCoA) carried out on 20 populations of *P. simplex* showed most dispersion within the 2*x* population and the mixed 2*x*–4*x* (Figure 2, adapted from Brugnoli et al., 2013 and Brugnoli et al., 2014). On the other hand, the tetraploid and mixed 4*x*–6*x* populations showed very limited dispersion, as is the case of the tetraploid populations from Corrientes and Villa Ana, where practically all individuals were located at the same point, indicating reduced intra-population variation (Figure 2). Most diversity was present among 4*x* populations. No correlation was observed between genetic and geographical distances.

Both species have similar mechanisms of generating variability in apomicts, so the search and conservation of such variability should be carried out in mixed populations (2*x*–4*x*) or in zones of contact between diploids and tetraploids. Additionally, individual plant collections from diverse locations with contrasting environments should be used as more diversity is expected among the apomictic genotypes from different locations.

### *Paspalum vaginatum* and *P. distichum*

*Paspalum vaginatum* Sw. is a high-quality turf mainly used in the coastal regions across the tropics and subtropics, and *P. distichum* L. is a wild relative native to South America. Both species belong to the Disticha group of *Paspalum*. They are differentiated because *P. distichum* has spikelets with pubescent glumes, whereas *P. vaginatum* has glabrous glumes (Chase, 1929). However, the identification of both species usually generates confusion because the type specimen for *P. distichum* contained pieces of both *P. distichum* and *P. vaginatum* (Eudy et al., 2017). *P. vaginatum* has typically been described as an allogamous sexual diploid (Bashaw et al., 1970) but later on a tetraploid cytotype was also reported (Hojsgaard et al., 2009). In contrast, *P. distichum* has been mentioned as a polyploid species with tetraploid and hexaploid cytotypes, including pentaploids and hyperpentaploids (Quarin and Burson, 1991; Echarte et al., 1992; Echarte and Clausen, 1993; Hojsgaard et al., 2009; Rua et al., 2010).

Eudy et al. (2017) demonstrated the characterization of 90 diploid and seven polyploid accessions belonging to the Disticha group of *Paspalum* by selecting seven SSR markers that differentiated closely related lines. They found many lines with identical SSR profiles, which were part of a group of individual selections made at the same time from golf courses or in natural areas. On the other hand, five genotypes from two germplasm banks (USDA and UGA) that were thought to be clonally identical had different SSR profiles. These results support the idea that genotyping material immediately after collection, storing this DNA fingerprint, and periodically reconfirming identity may be an appropriate approach to maintain plant diversity and avoid duplications in germplasm banks (Eudy et al., 2017). The authors recommended further plant collections in native South America habitats to increase diversity in the USA germplasm conservation system. Although they suggested vegetative plant collections, seed collections should be more appropriate since *P. vaginatum* is a cross-pollinated species.

### *Paspalum dilatatum*

*Paspalum dilatatum* Poir (Dallisgrass) is a forage species widely distributed in the subtropical and temperate regions around the world. It belongs to the informal subgeneric Dilatata group and is a multiploid species of hybrid origin with sexual tetraploid, apomictic pentaploid, and hexaploid cytotypes (Bashaw and Holt, 1958; Burson et al., 1991; Evers and Burson, 2004). Seven biotypes have been described for *P. dilatatum*: three are sexual tetraploid (*P. dilatatum* ssp. *flavescens*, *P. dilatatum* “Virasoro”, and *P. dilatatum* “Vacaria”), one is apomictic pentaploid (*P. dilatatum* ssp. *dilatatum*), and three are apomictic hexaploid (*P. dilatatum* “Chirú”, “Uruguaiana”, and “Torres”) (Burson et al., 1991; Evers and Burson, 2004; Williams et al., 2011). The predominant biotype is the apomictic pentaploid, which is widespread in South America and naturalized in other parts of the world (Evers and Burson, 2004).

An evolutionary analysis in the Dilatata informal group of *Paspalum* was carried out with microsatellite markers to clarify the relationships among biotypes and evolutionary pathways (Speranza, 2009). A clear genetic differentiation in nuclear microsatellite loci was observed between sexual tetraploid biotypes of the Dilatata group, which supports the hypothesis proposed by Burson (1983) that there is an independent origin of at least some of these biotypes. On the other hand, the low gene flow observed between the tetraploid biotypes maintains their genetic identity (Speranza, 2009). Two sexual tetraploid biotypes, *Paspalum dilatatum* ssp. *flavescens* and “Virasoro”, showed a high level of homozygosity for all microsatellites loci evaluated. Otherwise, the sexual tetraploid biotype “Vacaria” showed a greater number of polymorphic loci according to its lower degree of autogamy. Most apomictic biotypes seemed to have a uniclonal origin with very rare sexual recombination. Speranza (2009) suggested that the mechanisms for the formation of apomictic genotypes involve either unreduced female gametes or euploid pollen grains from the pentaploid biotype.

These results suggest that sexual tetraploid germplasm should be collected to include the greatest diversity. Single plant collections at each location may be appropriate since the sexual tetraploid germplasm behaves as autogamous.

### *Paspalum scrobiculatum*

*Paspalum scrobiculatum* L. (kodo millet) is one of the few *Paspalum* species native to the Old World tropics and used as cereal in India (Clayton, 1975; de Wet et al., 1983). This species has a particular genetic system within the *Paspalum* genus. It is a multiploid species with 4*x*, 6*x*, 8*x*, 10*x*, and 12*x* cytotypes. While the tetraploid is sexual the hexaploid and decaploid are diplosporous apomictic, with some potential for apospory in the hexaploid cytotypes (Bashaw et al., 1970; Pritchard, 1970; Chao, 1974; Ma et al., 2009). Chao, (1974) observed regular meiosis and sexual reproduction, with potential for apomixis due to the presence of aposporous embryo sacs, for the 12*x* cytotype.

A diversity analysis on morphological traits was performed for some germplasm collections of *P. scrobiculatum*. Dhagat (1978) studied the genetic diversity among 6 exotic and 90 indigenous germplasm accessions of *P. scrobiculatum*. Morphological and physiological traits, such as days to 50% flowering and maturity, plant height, and straw yield were the most important traits for the differentiation among the germplasm accessions from different geographical regions. Parihar (1985) found that quantitative traits such as plant height, tillers/plant, flag leaf length, peduncle length, and ear length showed the maximum percentage contribution to genetic divergence of 100 genotypes of *P. scrobiculatum*.

Formation of a core collection is an important strategy to enhance use of diverse germplasm with agronomically beneficial traits in applied breeding (Upadhyaya et al., 2014). A germplasm collection of kodo millet from India composed of 656 accessions was evaluated for 20 morphoagronomic traits. A core subset of 75 accessions (∼11%) was selected from the germplasm collection maintained in the ICRISAT genebank, Patancheru, India. These core collections are ideal genetic resources to identify new sources of variation to be used in crop improvement (Upadhyaya et al., 2014).

### Other *Paspalum* Species

Plicatula is an informal taxonomic subgeneric group within *Paspalum* (Chase, 1929) with a great diversity of species, biotypes, and forage attributes (Novo et al., 2017). This group includes approximately 30 species, most of which are tetraploid and apomictic (Ortiz et al., 2013). *Paspalum plicatulum* has sexual, self-sterile diploid, and apomictic tetraploid cytotypes, while *P. atratum* Swallen, *P. guenoarum* Arechav., and *P. nicorae* Parodi have only apomictic tetraploid cytotypes (Ortiz et al., 2013).

Microsatellite markers were evaluated in different species of *Paspalum* conserved in Brazil. Some species of the Plicatula group, such as *P. atratum* and *P. plicatulum*, showed a high intraspecific genetic variability, but there was no clear distinction among different species (Cidade et al., 2013). This lack of differentiation among the different taxa of the Plicatula group was attributed to the genetic similarity of these species as well as interspecific hybridization throughout their evolution (Cidade et al., 2013). Many species of the Plicatula group are morphologically variable (Oliveira, 2004) and this variation could be obtained and conserved from accessions collected at different environments.

*Paspalum buckleyanum* Vasey (syn. *P alcalinum* Mez) is a wild forage species adapted to rangelands with alkaline and saline soils. It is a multiploid species with sexual self-fertile diploid, facultative apomictic tetraploid, obligate apomictic pentaploid, hexaploid and heptaploid cytotypes (Burson, 1997; Hojsgaard et al., 2009; Sartor et al., 2011). A genetic variability analysis with AFLP markers in three hexaploid populations of *P. buckleyanum* showed all individuals of the same population had identical genetic profiles, indicating that a single genotype predominated in each population (Sartor et al., 2013). Single-plant collections at each site may be the best approach for increasing the conserved diversity for *P. buckleyanum* since the hexaploid forms monoclonal populations.

*Paspalum denticulatum* and *P. rufum* are also multiploid species with sexual diploids and apomictic polyploids (Siena et al., 2008; Sartor et al., 2011). Pure tetraploid populations of *P. denticulatum* behaved as facultative apomicts with a low level of variation in comparison to the mixed (2*x*–4*x*) population (Sartor et al., 2013). Diploid and diploid-tetraploid mixed populations of *P. rufum* exhibited greater genetic diversity than those observed in uniformly tetraploid population. However, the level of variability in pure tetraploid populations of *P. rufum* was observed to be greater than that in the tetraploid populations of *P. denticulatum*. The levels of genetic and genotypic variability in diploid and mixed populations of *P. denticulatum* and *P. rufum* may be mainly due to the reproductive system of diploid members and the gene flow from diploids to polyploids (Sartor et al., 2013). In both species, collection of diversity would be most efficient by focusing on obtaining many individuals or seed from mixed 2*x*–4*x* populations.

## Germplasm Conservation

Plant genetic resources are essential for sustainable agricultural production. Germplasm banks are centers for conservation of genetic resources under appropriate conditions that permit to prolong their viability and availability. There are 1,750 germplasm banks in the world which include nearly 7.5 million accessions belonging to cultivated species, wild and local varieties of importance to humans (food crops) and livestock (forages) (FAO, 2014).

There are two main and complementary methods to conserve genetic resources, i.e., *ex situ* and *in situ*. *Ex situ* conservation is characterized by preserving the genetic resources off-site (Plucknett et al., 1987). According to Gepts (2006), 90% of these genetic resources are conserved as seeds in cold storage at low relative humidity (3–7%). Germplasm can also be conserved as living plants in field gene banks, particularly when the species are sterile or produce recalcitrant seeds, which are those that can not survive desiccation without loss of viability. Germplasm can also be maintained *in vitro* (in test tubes on plant nutrient medium). More specialized and technically intensive methods are being used or investigated such as cryopreservation (liquid nitrogen: -196°C), artificial seeds, pollen, and DNA (FAO, 2014). The second method of conservation is *in situ* (on-site). This type of conservation can take place in farmers’ fields (for cultivated crop) or in natural environments (for wild relatives of crop plants or wild species). Comparing both conservation methods, the advantages of *ex situ* conservation are the capacity of storing a large number of accessions, the facility of access to the germplasm for characterization, evaluation and distribution, and secure conservation conditions. However, *in situ* conservation is used because landraces are an important component of indigenous cultures, it allows evolution to proceed, its cost is low, and it is the primary form of conservation for wild crop relatives (Gepts, 2006).

Most *Paspalum* germplasm collections have been conserved *ex situ* in 11 gene banks around the world (Table 1). This germplasm is mainly conserved as seeds at low temperature and low humidity. However, living plants are also preserved in a few gene bank collections. The National Bureau of Plant Genetic Resources (NBPGR), India, contains the largest number of accessions, but all belonging to *P. scrobiculatum* (kodo millet). U.S. National Plant Germplasm System (USDA), EMBRAPA (Brazil) and IBONE (Argentina) germplasm banks preserved the greatest number of species with 48, 51, and 72, respectively.

**Table 1:** Germplasm banks of *Paspalum* species in different parts of the world.

Germplasm bank	Country	N° accessions	N° species	Best represented species	Reference
National Bureau of Plant Genetic Resources (NBPGR)	India	2,273	1	*P. scrobiculatum*	Hariprasanna (2017)
All India Coordinated Small Millets Improvement Project (AICSMIP)	India	1,538	1	*P. scrobiculatum*	http://www.millets.res.in/aicrp_small.php
U.S. National Plant Germplasm System	USA	1,242	48	*P. scrobiculatum, P. dilatatum, P. notatum*	https://npgsweb.ars-grin.gov/gringlobal/search.aspx
Svalvard Global Seed Vault	Norway	907	32	*P. scrobiculatum, P. notatum P. floridanum*	https://www.nordgen.org/sgsv/
EMBRAPA	Brazil	746	51	*P. urvillei, P. dilatatum, P. plicatulum*	http://alelobag.cenargen.embrapa.br/AleloConsultas/Passaporte/taxonomia.do
ICRISAT Genebank	India	665	1	*P. scrobiculatum*	http://genebank.icrisat.org/
Germplasm bank of FCA-UNNE and IBONE	Argentina	434	72	*P. notatum, P. dilatatum, P. simplex*	This work
Margot Forde Forage Germplasm	New Zealand	373	36	*P. dilatatum, P. plicatulum, P. notatum*	https://www.agresearch.co.nz/about/our-subsidiaries-and-joint-ventures/margot-forde-forage-germplasm-centre/
Australian Pastures Genebank	Australia	301	33	*P. notatum, P. nicorae, P. paniculatum*	https://apg.pir.sa.gov.au/gringlobal/search.aspx
CIAT	Colombia	156	18	*P. plicatulum, P. scrobiculatum, P. conjugatum*	https://ciat.cgiar.org/what-we-do/crop-conservation-and-use/tropical-forage-diversity/
Genetic Resources Center, NARO	Japan	114	5	*P. notatum, P. dilatatum, P. scrobiculatum*	https://www.gene.affrc.go.jp/databases-plant_search_en.php

An important germplasm bank of *Paspalum* species exists in Argentina. This gene bank includes 434 accessions from 72 species. These were collected in different regions of the natural distribution of the species. These materials are preserved mainly as seed, but are also conserved as plants in greenhouses or grown in the field (Table 2). The species represented by the greatest number of accessions are: *P. notatum* (77), *P. dilatatum* (41), and *P. simplex* (34), which represent 35% of the total number of accessions preserved in this gene bank.

**Table 2:** Germplasm conserved at Facultad de Ciencias Agrarias, Universidad Nacional del Nordeste, and Instituto de Botánica del Nordeste, Corrientes, Argentina.

Species	Accessions			
	Total number	Conserved		
		Seed	Plant	
			Greenhouse	Field
*Paspalum acuminatum* Raddi	2	2	0	0
*P. alcalinunm* Mez (=*P.buckleyanum* Vasey)	11	11	2	0
*P. almum* Chase	1	1	0	0
*P. arundinellum* Mez	7	7	0	4
*P. atratum* Swallen	10	10	1	2
*P. chacoense* Parodi	1	1	1	0
*P. chaseanum* Parodi	3	3	3	0
*P. commune* Lillo	2	1	1	0
*P. compressifolium* Swallen	4	3	0	2
*P. conduplicatulum* Canto-Dorow, Valls & Longhi-Wagner	1	1	0	0
*P. conjugatum* P. J. Bergius	3	3	0	2
*P. conspersum* Schrad.	2	2	0	1
*P. cromyorrhizon* Trin.ex Döll	3	3	0	3
*P. dasypleurum* Kunze ex Desv.	4	4	3	0
*P. dedeccae* Quarin	1	1	0	1
*P. densum* Poir.	1	1	0	0
*P. denticulatum* Trin.	6	6	2	1
*P. dilatatum* Poir	41	39	6	7
*P. durifolium* Mez	10	10	0	3
*P. equitans* Mez	1	1	0	1
*P. erianthum* Nees ex Trin.	1	0	0	1
*P. exaltatum* J. Presl	2	0	1	2
*P. falcatum* Nees ex Steud.	3	0	3	0
*P. fimbriatum* Kunth	2	2	0	0
*P. glaucescens* Hack.	1	1	1	0
*P. guenoarum* Arechav.	12	12	3	5
*P. haumanii* Parodi	3	1	0	2
*P. humboldtianum* Flüggé	3	1	2	1
*P. indecorum* Mez	3	2	3	0
*P. intermedium* Munro ex Morong & Britton	10	10	0	2
*P. ionanthum* Chase	4	4	0	4
*P. jesuiticum* Parodi	1	1	0	1
*P. kempffii* Killeen	1	1	0	1
*P. laxum* Lam.	1	1	0	0
*P. lenticulare* Kunth	23	23	1	10
*P. lilloi* Hack.	1	0	1	0
*P. lividum* Trin.	1	0	1	0
*P. macedoi* Swallen	1	1	0	0
*P. malacophyllum* Trin.	13	13	5	9
*P. mandiocanum* Trin.	1	1	0	0
*P. millegrana* Schrad.	2	0	1	1
*P. minus* E. Fourn.	1	1	0	0
*P. modestum* Mez	2	2	0	1
*P. nicorae* Parodi	6	5	2	2
*P. notatum* Flüggé	77	55	10	72
*P. oteroi* Swallen	3	2	0	3
*P. ovale* Nees ex Steud.	2	2	0	1
*P. palustre* Mez	1	1	0	1
*P. paniculatum* L.	2	2	0	2
*P. pauciciliatum* (Parodi) Herter	2	1	1	0
*P. paucifolium* Swallen	1	0	0	1
*P. plenum Chase*	1	0	0	1
*P. plicatulum* Michx.	18	18	1	10
*P. procurrens* Quarin	4	4	3	2
*P. pubiflorum* Rupr. ex E. Fourn.	1	1	0	0
*P. pumilum* Nees	1	1	1	0
*P. quadrifarium* Lam.	4	2	0	3
*P. quarinii* Morrone & Zuloaga	4	0	0	4
*P. redondense* Swallen	2	0	2	0
*P. regnellii* Mez.	6	6	0	1
*P. remotum* J. Rémy	2	2	0	0
*P. rojasii* Hack.	1	1	1	1
*P. rufum* Nees ex Steud.	12	4	0	12
*P. simplex* Morong ex Britton	34	34	0	5
*P. umbrosum* Trin.	1	0	0	1
*P. unispicatum* (Scribn. & Merr.) Nash	4	4	2	3
*P. urvillei* Steud.	19	19	0	2
*P. usterii* Hack.	5	3	0	5
*P. vaginatum* Sw.	5	0	6	1
*P. virgatum* L.	2	1	0	1
*P. volcanensis* Zuloaga, Morrone & Denham	2	2	1	1
*P. wrightii* Hitchc. & Chase	6	2	0	6
**Total**	**434**	**359**	**71**	**208**

Apomixis, present in a large number of *Paspalum* species and accessions, allows for conserving polyploid and highly heterozygous genotypes through seed instead of live plants. This advantage increases the conservation efficiency taking into account that a larger number of accessions can be conserved at lower cost. Additionally, this germplasm is conserved with lower risk of disease transmission as compared with asexual generations by vegetative propagation. The main disadvantage of apomixis for germplasm conservation relates to the fact that only for some species it is possible to access the diversity contained in apomictic genotypes through hybridization (Kumar et al., 2017). The availability of compatible sexual germplasm is the key for hybridizing apomictic accessions (Miles, 2007; Kumar et al., 2013).

Since polyploidy and apomixis predominate in *Paspalum*, the possibility of transferring the natural diversity present in apomictic genotypes to sexual synthetic tetraploid populations may be crucial for utilizing the germplasm. Zilli et al. (2018) have described a novel breeding approach developed for *P. notatum* that allow to transfer the diversity present in a group of apomictic ecotypes distributed throughout the Americas to a single synthetic tetraploid population. A similar approach is being used in the Plicatula group of *Paspalum*, which includes near 30 species (Novo et al., 2017). These populations are expected to contain a high level of diversity and can be interesting for preserving the genetic diversity in species and groups of related species within the genus.

## Genetic Improvement

### Impact of Apomixis: Inheritance, Expressivity, and Early Identification of Apomicts

Polyploidy is present in around 75% of *Paspalum* species, ranging from triploid to 12-ploid, tetraploidy being the most common polyploid type (Ortiz et al., 2013). Polyploidy and apomixis are strongly related in *Paspalum*, and most polyploid cytotypes reproduce by apomixis. The studies on apomixis and its genetic control have been of great interest in recent decades, mainly because of the interest in transferring apomictic reproduction to the major economic crops; however, transfer of apomixis to crop species has not been successful mainly because the gene(s) involved have not yet been identified (Kumar et al., 2019). One of the objectives of manipulating apomixis for breeding purposes relates with the possibility of fixing heterosis in F_1_ for the traits of interests (Hanna, 1995; Miles, 2007; Kumar et al., 2017). The superiority of apomictic hybrids is expected to be retained across the reproductive cycles.

The generation of sexual tetraploid individuals by chromosome doubling of sexual diploids in *P. notatum* (Burton and Forbes, 1960; Quarin et al., 2001; Quesenberry et al., 2010), *P. simplex* (Cáceres et al., 1999), and *P. plicatulum* (Sartor et al., 2009) allowed the generation of segregating populations by means of hybridization between sexual and apomictic tetraploids. Martínez et al. (2001) used F_1_, F_2_ and back-crosses to study the inheritance of apospory in *P. notatum*. The authors reported that apomixis was inherited as a major dominant factor with distorted segregation in favor of sexual individuals, probably due to some pleiotropic lethal effect of the major gene(s) or partial lethality factors linked to the apospory locus. Recent cytogenetic studies demonstrated meiotic abnormalities associated with apospory in *P. notatum* (Dahmer et al., 2008; Podio et al., 2012) supporting this hypothesis. Later, Martínez et al. (2007) reported that apospory could not be transferred by monoploid male gametes (*n* = *x*), supporting the hypothesis postulated by Nogler (1984) that apomixis can only be transferred under heterozygous conditions. In addition, Aguilera et al. (2015) reported distorted segregation in favor of sexual inter-specific hybrids of crosses between an induced sexual tetraploid *P. plicatulum* and an apomictic *P. guenoarum*. Interestingly, when back-crossing the induced sexual tetraploid plant of *P. plicatulum* with apomictic F_1_ hybrids, no distortion was observed in the segregation patterns.

Apomictic reproduction generally does not imply the exclusive generation of clonal progenies; facultative apomictic reproduction is observed in almost all species of *Paspalum* studied to date. In the case of apomictic hybrids of *P. notatum*, the expressivity of apospory is more variable than in natural apomictic ecotypes, ranging from 1 to 100% (Martínez et al., 2001; Acuña et al., 2009; Acuña et al., 2011; Zilli et al., 2015; Marcón et al., 2019). The causes of the variation in apospory expressivity in the progeny of sexual × apomictic crosses remain uncertain, but some hypothesis have been proposed. For instance, Nogler, (1984) postulated the timing of apospory induction as the cause of variable expressivity of apospory in apomictic hybrids of *Ranunculus sp.*; if the apospory induction occurs prior to meiosis the result would be an aposporic embryo sac, otherwise, it would be sexual. The author observed that apospory expressivity in inter-specific hybrids of *Ranunculus* decreases when back-crossing with the female parent. This hypothesis would explain the results reported by Acuña et al. (2009, 2011) in apomictic hybrids of *P. notatum*, where the proportion of highly apomictic hybrids decreases in the progeny when crossing first and second generation apomictic hybrids × sexual genotypes. Zilli et al. (2015) reported that hybrids of *P. notatum* exhibited low or high apospory expressivity but a reduced proportion of hybrids exhibited intermediate levels of expressivity. Therefore, the variable expressivity may be related to a single major gene. In addition, temporal variation in the level of expressivity of apomixis has been reported in several *Paspalum* species. For instance, Quarin (1986) reported variable apospory expressivity in *P. cromyorrhizon*, attributing the phenomenon to variation in the photoperiod; however, the authors indicated that other environmental factors such as temperature and water stress could have a significant effect on apospory expressivity. Burton (1982a) reported that photoperiod and water and nitrogen deficit did not produce a shift from apomictic to sexual reproduction in natural apomictic *P. notatum* plants. However, the methodology used to determine apomixis expressivity, by evaluation of the homogeneity of the progeny, would not be enough sensitive to detect small changes in expressivity. Rios et al. (2013a) reported that higher apospory expressivity in *P. notatum* is observed during summer, whereas the expressivity is lower during spring and fall, attributing this variation to photoperiod. The mechanism(s) involved in the variation for apospory expressivity remains unclear, and due to its substantial importance for breeding not only apomictic grasses, but also main apomictic crops in the future, further research should be addressed to this topic.

Determining the mode of reproduction and expressivity of apomixis have been important for breeding programs. Progeny test was the first technique adopted in the genus *Paspalum*. This technique is based on evaluation of the uniformity of the offspring and its identity to the maternal plant. It was used for determining mode of reproduction and expressivity of apomixis in *P. notatum* (Burton, 1948; Burton, 1982a; Ortiz et al., 1997; Rebozzio et al., 2011). It is a reliable technique because of the direct observation on the progeny, but is a time-consuming and demanding method as it requires to grow a large number of progenies in the field, and having the limitation of a fewer morphological markers available. The use of DNA-based markers may overcome the limitations (Chandra et al., 2010; Yadav et al., 2019). The generation of linkage maps and identification of genomic regions in the genus *Paspalum* (Pupilli et al., 2001; Labombarda et al., 2002; Martínez et al., 2003; Pupilli et al., 2004; Stein et al., 2004; Stein et al., 2007), allowed the identification of the apomixis-controlling genomic region. These kinds of studies allowed the development of molecular markers linked to apospory in *P. notatum* (Pupilli et al., 2001; Martínez et al., 2003; Pupilli et al., 2004; Stein et al., 2004; Stein et al., 2007, Rebozzio et al., 2012) and *P. simplex* (Labombarda et al., 2002). The availability of molecular markers allows breeders to achieve early classification of reproductive mode in segregating progenies, saving time and resources (Zilli et al., 2015; Brugnoli et al., 2019). Due to variable apospory expressivity reported in *Paspalum* species, this technique does not allow identification of highly apomictic hybrids, therefore auxiliary techniques such as mature embryo sac observation, flow cytometry, or progeny tests, using morphological or molecular markers, are needed. Mature embryo sac observation has been extensively used for determining mode of reproduction and apospory expressivity in *P. notatum* (Ortiz et al., 1997; Martínez et al., 2001; Quarin et al., 2001; Acuña et al., 2007; Martínez et al., 2007; Acuña et al., 2009; Acuña et al., 2011; Rebozzio et al., 2011; Zilli et al., 2015; Zilli et al., 2018), *P. cromyorrhizon* (Quarin, 1986), *P. malacophyllum* (Hojsgaard et al., 2016), *P. rufum* (Siena et al., 2008; Delgado et al., 2014; Soliman et al., 2019), among others. This technique was developed by Young et al. (1979) and recently modified by Zilli et al. (2015); it requires plants at flowering stage and provides reliable information about expressivity of apomixis (Ortiz et al., 1997) and also is inexpensive, rapid, and straightforward (Zilli et al., 2018). Flow cytometry on seeds was used in *P. notatum* (Urbani et al., 2017), *P. simplex* (Brugnoli et al., 2014), *P. malacophyllum* (Hojsgaard et al., 2016), *P. rufum* (Delgado et al., 2014), and in inter-specific hybrids of the Plicatula group of *Paspalum* (Aguilera et al., 2015). This technique provides similar information to embryo sac observation, but determining expressivity of apomixis at seed stage, though giving a more accurate approximation to what is expected in the progeny, comes at a higher cost in comparison to embryo sac observations. Therefore, molecular markers linked to apospory could be used for determining mode of reproduction at seedling stage, planting in the field only the apomictic ones, and mature embryo sac observation or flow cytometry on seeds could be used to identify and select highly apomictic hybrids for further evaluations, saving time and resources. Identification of obligate apomictic hybrids is a key factor for breeding programs to ensure genetic stability through the successive reproductive cycles, which is the most important factor for cultivar development.

### Breeding Methods For Diploid and Polyploid Germplasm

#### Ecotype Selection

The most common breeding approach adopted for warm-season grasses in the last century was the selection within the natural variability present in the species (Gates et al., 2004). This method was adopted from vegetatively propagated species and consists of selection of ecotypes from the natural distribution area of the species, selecting among germplasm bank introductions or landraces. Ecotypes are evaluated in multiple locations and years for forage and/or grain production and quality, or turf performance, in addition to tolerance for biotic and abiotic stress. This breeding method is particularly useful for apomictic species due to the possibility of clonal reproduction by seeds (Gates et al., 2004). However, this methodology may be suitable to accomplish the short and midterm objectives as no novel variability is created (Zilli et al., 2018). Despite this, the majority of the released cultivars of *Paspalum* were selected using this breeding approach.

#### Hybridization

Two hybrid cultivars were released for diploid *P. notatum*: Tifhi 1 (Hein, 1958) and Tifhi 2. These two diploid hybrids were selected by considering the specific combining ability of pairs of self-incompatible genotypes (Burton, 1984). These hybrids exhibited greater forage yield and liveweight gain than the cultivar Pensacola. However, their adoption by farmers was not significant because of the high cost of seed production (Burton, 1984).

The general idea of using hybridization in apomictic plants relates to the possibility of releasing the natural diversity present in apomictic ecotypes and fixing superior F_1_ hybrids. These novel apomictic hybrids are expected to be genetically stable through indefinite asexual generations (Miles, 2007). Apomixis involves the formation of a chromosomally unreduced embryo from a megaspore mother cell or a somatic cell; however, these plants produce genetically recombined and chromosomally reduced male gametes (Hanna, 1995). Therefore, an apomictic plant could be used as male parent when a sexual compatible plant of same ploidy level is available (Kumar et al., 2017). The efficiency of crosses is expected to be greater when parents have the same ploidy level and number of chromosomes. In a few *Paspalum* species, sexual tetraploid genotypes were generated by chromosome doubling of sexual diploids. Induced sexual 4*x* plants of *P. notatum*, like their diploid progenitors, were allogamous due to self-incompatibility. Burton et al. (1970) indicated that self-incompatibility may be the result of the S-Z mechanism, commonly observed in grasses.

Several attempts had been made to obtain highly heterotic apomictic hybrids by crossing sexual × apomictic genotypes. Promising hybrids were generated in *P. notatum* (Burton, 1992; Acuña et al., 2009; Acuña et al., 2011; Zilli et al., 2015). Burton (1992) described the generation of four segregating families obtained by crossing three induced tetraploid sexual plants × two apomicts. One of these families exhibited greater forage and seed yield and was informally named Tifton 54. Acuña et al. (2009) obtained 591 hybrids by crossing 11 experimental sexual tetraploid plants × 5 apomicts. Several hybrids exhibited heterosis for agronomic and morphological traits; however, the proportion of highly apomictic hybrids in the progenies was low (11%). In addition, the autors reported that sexual hybrids behaved as self-compatible like their apomictic parents, and unlike the self-incompatible diploid and induced sexual tetraploids. Acuña et al. (2011) generated 2,700 hybrids by crossing 11 sexual F_1_ hybrids × 5 apomictic F_1_ hybrids and the apomictic cultivar Argentine. The autors reported the occurrence of heterosis for all agronomic and morphological traits evaluated. The proportion of highly apomictic hybrids was lower (3%) than observed by Acuña et al. (2009). Zilli et al. (2015) generated 524 hybrids crossing three experimental sexual plants × nine apomicts. Variable levels of heterosis were observed depending on the parental combination and the evaluated trait. The authors observed that segregation for mode of reproduction depended on the male parent used. However, the proportion of highly apomictic hybrids was low (8%). The low proportion of highly apomictic hybrids is one of the most important obstacles to the generation of superior apomictic hybrids. Finally, the first apomictic hybrid cultivar of *P. notatum* was recently released commercially (Urbani et al., 2017).

Recently, Brugnoli et al. (2019) generated 232 hybrids of *P. simplex* by crossing two induced sexual plants × seven apomicts. The average proportion of apomictic hybrids was 1:2.4 (apomictic:sexual hybrids), but was variable and depended on the parental combination. In addition, the authors reported no difference in agronomic and morphologic traits between sexual and apomictic hybrids. Several apomictic hybrids combined agronomic traits of interest and will be further evaluated.

The use of hybridization between related species is a suitable approach for breeding in the genus *Paspalum*. Inter-specific hybrids were generated in the Plicatula group of *Paspalum* using a chromosome-doubled sexual plant of *P. plicatulum* and apomictic ecotypes of different species of the Plicatula group (Novo et al., 2013; Pereira et al., 2015; Novo et al., 2016; Novo et al., 2017). New highly apomictic hybrids were generated, and several were characterized as superior for forage yield, cold tolerance, and cattle preference with respect to the apomictic male parent (Da Costa Huber et al., 2016; Motta et al., 2017; Novo et al., 2017). This approach may also be used to hybridize *P. vaginatum* and *P. distichum*, since *P. vaginatum* is sexual and cross-pollinated and *P. distichum* is mainly tetraploid and apomictic (Bashaw et al., 1970; Ortiz et al., 2013). The generation of an induced sexual tetraploid plant will be needed, but this method may facilitate the creation of genetically uniform seeded turf cultivars.

Another hybridization approach was used attempting to increase ergot (*Claviceps paspalli*) resistance in *P. dilatatum*. The widely distributed forage *P. dilatatum* is apomictic and pentaploid, and is highly susceptible to ergot. *P. urvillei* is a closely related species but is tetraploid and sexual. Caponio and Quarin (1990) generated inter-specific hybrids crossing a sexual tetraploid ecotype of *P. dilatatum* and a sexual tetraploid genotype of *P. urvillei*. These hybrids were back-crossed to *P. dilatatum* and evaluated for tolerance to ergot and seed yield (Schrauf et al., 2003); one of these hybrids was selected for superior performance and released as a cultivar namely “Primo” (INASE, 2013).

Molecular markers have been useful for hybridization in apomictic *Paspalum* species. Molecular markers linked to apospory were used for early identification of aposporic hybrids in *P. notatum* (Zilli et al., 2015; Zilli et al., 2018; Marcón et al., 2019) and *P. simplex* (Brugnoli et al., 2019). In addition, molecular markers were used to assess the efficiency of crossing methods (Aguilera et al., 2015; Zilli et al., 2015; Novo et al., 2017; Brugnoli et al., 2019). Random molecular markers (ISSR and SSR) were sucessfully used to predict the segregation for mode of reproduction and heterosis for forage yield in *P. notatum* (Marcón et al., 2019). The greater the genetic distance between parents, the greater the fraction of apomictic hybrids within the progeny and the level of heterosis for forage yield, indicating the advantage of crossing unrelated parents.

#### Recurrent Restricted Phenotypic Selection (RRPS)

Mass selection is a useful method for self- and open-pollinated sexual species, suitable for highly heritable traits. After the development of an efficient technique for hybridizing *P. vaginatum*, vegetatively propagated F_1_ hybrids are selected from segregating families (Hanna et al., 2013). Selected F_1_ hybrids may be part of the next cycle of crosses and selection. Recently, new techniques for hybridizing *P. scrobiculatum* were developed leading to the generation of large segregating families (Hariprasanna, 2017); therefore, mass selection may be suitable for improving this species. Moreover, the adoption of the breeding schemes will also depend on the availability of resources in breeding programs.

Restricted recurrent phenotypic selection (RRPS) is mass selection on which restrictions are imposed in order to increase its efficiency; this method was used by Burton (1974) for improving diploid sexual germplasm of *P. notatum*. The author applied restrictions such as the use of grids including 25 plants each, inter-crossing the selected plants instead of using open pollination (doubling genetic gain because selection is imposed on both maternal and paternal progenitors), controlling the inter-crossing by bagging inflorescences, and shaking the bags at flowering. Selected plants were represented by two inflorescences, and highly self-sterile plants reduced likelihood of selfing. Burton (1982b) proposed some modifications to this method, discarding progenies based on the performance of maternal progenitors, and improving cultural practice to achieve flowering during the first growing period. These modifications allowed using a 1-year selection cycle instead of two, and achieving a genetic gain four times greater than conventional mass selection, saving time and resources. Diploid cultivars Tifton 9, TifQuik and UF-Riata of *P. notatum* were obtained using this selection methodology (Blount and Acuña, 2009).

Due to the increasing interest in breeding *P. vaginatum* as an eco-friendly turf, RRPS could be suitable for improving this species. In the case of breeding programs focused on turf cultivars, as in the case of *P. vaginatum* and some *P. notatum*, the breeding scheme and selection methodology should be adjusted according to the propagation method (seed or vegetative) of the new cultivar (Hanna et al., 2013). In addition, RRPS would also be appropriate for improving the sexual diploid and polyploid germplasm of other *Paspalum* species.

#### Use Of Synthetic Sexual Tetraploid Populations

The use of synthetic sexual tetraploid populations as base population in breeding programs focused on apomictic species and adopting the RRPS for breeding these populations was first proposed for *P. notatum* (Burton, 1992). A mostly sexual tetraploid population was established in southern USA in 1974. Five cycles of RRPS were conducted, achieving improvement in individual plant biomass yield; but then, the program was discontinued. Another attempt was made in 1983, where two populations, obtained using different crosses, were established. After three cycles of selection 28 plants were identified as superior, and one of them, hybrid #7 produced more forage than cultivar Argentine and showed greater ergot resistance and seed yield. However, no cultivars were released from these populations and the program was discontinued. The lack of success was probably due to the limited understanding of the inheritance of apomixis at the time (Miles, 2007).

**Figure 3:**
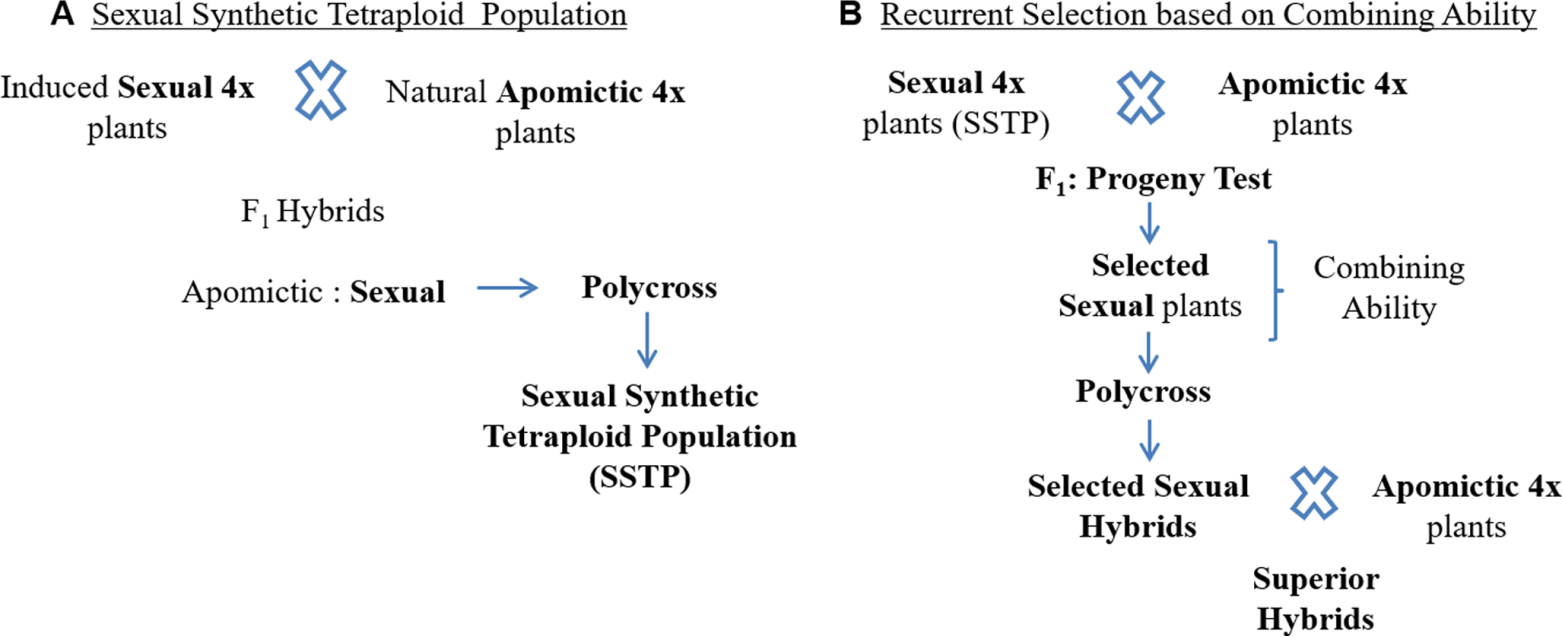
**(A)** Generation of a sexual synthetic tetraploid population (SSTP) of *Paspalum notatum* from crosses between a few experimental sexual tetraploid plants and a group of natural apomictic tetraploid plants. **(B)** Recurrent selection based on combining ability applied to improve a group of sexual genotypes belonging to a SSTP.

Recently, Zilli et al. (2018) generated a synthetic sexual tetraploid population of *P. notatum* composed of 306 plants by crossing three experimental sexual tetraploid genotypes with 10 natural apomictic genotypes and intercrossing 29 sexual F_1_ hybrids (Figure 3A). The 10 apomictic male parents were selected to represent the natural distribution area of the species, and to transfer the genetic variability from the apomictic germplasm to the sexual. In addition, this population was characterized for mode of reproduction, fertility, and ploidy level. It constitutes a base population for breeding. Furthermore, Zilli et al. (2019) estimated the genetic variability of this population and its ancestors by molecular markers, agronomic and morphologic traits, and seed fertility, and determined that the genetic variability contained in the apomictic germplasm was effectively transferred to the sexual synthetic germplasm. Additionally, the authors reported individuals from the sexual synthetic tetraploid population combining traits of interest for breeding programs. The availability of a sexual synthetic population allows breeders access to genetic variability in the sexual counterpart of apomictic × sexual crosses in breeding programs aimed to obtain highly productive apomictic hybrids. RRPS would be a suitable breeding method; however, due to the fact that improving apomictic species is focused on obtaining superior apomictic hybrids, the use of recurrent selection based on combining ability (Comstock et al., 1949) is expected to be more appropriate for accumulating additive as well as non-additive genetic effects (Figure 3B). In addition, this method allows the evaluation of the apomictic hybrids obtained from each test cross as potential new cultivars.

The generation of inter-specific hybrids in the Plicatula group of *Paspalum* (Novo et al., 2017) would allow the creation of a synthetic sexual tetraploid population for breeding and conservation purposes by inter-crossing selected F_1_ sexual hybrids. This breeding method could be also appropriate for improving other apomictic species, as was demonstrated in *Brachiaria* spp. (Miles and Escandón, 1997; Miles et al., 2006).

#### Genetic Transformation

Earlier plant breeding was restricted to the use of genes of the same or related species with different degrees of difficulty. The transfer of genes between unrelated species is not possible using conventional breeding approaches. Genetic engineering allows breeders to clone a gene from any organism and insert it into another organism, allowing the introduction of novel genetic variation for breeding (Vogel and Burson, 2004). Since the generation of the first genetically modified tall fescue (Wang et al., 1992), great progress was observed in many temperate forage species such as annual and perennial rye grass, red and tall fescue, and white clover, but only a few in warm-season species, were alfalfa received most of the attention (Wang and Brummer, 2012). Genetic transformation allows also the down- or up-regulation of specific genes (Wang and Brummer, 2012).

In the genus *Paspalum*, just two species have been used for genetic transformation, *P. notatum* and *P. dilatatum*. However, no transgenic cultivar has been released. Transgenic *P. notatum* plants have been generated using biolistic apparatus (Smith et al., 2002; Gondo et al., 2005; Agharkar et al., 2007; James et al., 2007; Luciani et al., 2007; Sandhu et al., 2007; Zhang et al., 2007; Giordano et al., 2014; Mancini et al., 2014; Muguerza et al., 2014). In these studies, phosphinothricin acetyltransferace (*bar* and *pat* genes) and neomycin phosphotransferace II (*npt2* gene) were used as selectable markers. Sandhu et al. (2007) reported that the transgenic *P. notatum* plants were resistant to gluphosinate ammonium under field and greenhouse trials. James et al. (2007) reported the transfer of a transcription factor from xeric *Hordeum spontaneum*, generating transgenic *P. notatum* plants tolerant to severe salt stress and dehydration under controlled environment conditions. Muguerza et al. (2014) were successful in obtaining low lignin content *P. notatum* plants by down-regulation of cinnamyl alcohol dehydrogenase gene expression. The authors reported that four out of nine transgenic plants exhibited a significant increment by 5.6 to 10.4% in the *in vitro* dry matter digestibility. A similar approach was used by Giordano et al. (2014) on *P. dilatatum* achieving a reduction of up to 20% on lignin content and an increment of up to 4% for *in vitro* dry matter digestibility. Mancini et al. (2014) developed a modified transformation method aiming to future evaluation of candidate genes for apomictic reproduction, which will be an important advance in the identification of the gene(s) responsible for apomictic reproduction and its potential transference to major crops.

Genetic transformation is a potentially useful tool for genetic improvement of forage and turf crops. However, its potential has been inhibited due to stringent regulation of transgenic plants, delaying the release and adoption of new cultivar because of concerns regarding pollen flow (Rios et al., 2017). However, (Sandhu et al., 2009; Sandhu et al., 2010) evaluated gene flow using glufosinate-resistant apomictic *P. notatum* plants as pollen donors and sexual diploids and apomictic tetraploids as pollen receptors placed between 0.5 and 3.5 meters apart. The authors reported a frequency of 0.03% of transgenic triploids or near-triploids in the progeny of sexual diploids, and less than 0.16% in the progeny of apomictic tetraploids. In addition, most of the triploids and near-triploids exhibited very low vigor and fertility. Therefore, ploidy level and apomictic reproduction act as barriers in pollen mediated gene transfer from transgenic to non-transgenic plants. The drawnbacks of this approach relates to the fact that any gene transfer to wild apomictic relatives would be very efficiently propagated over generations.

Mutagenesis and somaclonal variations are potentially useful tools for genetic improvement, overcoming the regulations imposed on transgenics. One of the first attempts in *Paspalum* was reported by Burton and Jackson (1962) using radiation breeding in apomictic prostrate *P. dilatatum var. pauciciliatum*. The authors reported the generation of plants mutanted for vegetative and floral trait, but no improvements for ergot resistance or seed quality and yield were found. In addition, the radiation treatment did not produce a shift from apomictic to sexual reproduction. Recently, Heckart et al. (2010) were successful in using in vitro selection to develop herbicide resistant plants of *P. vaginatum*. The authors used tissue culture looking for somaclonal variation, using sethoxydim (2-cyclohexen-1-one, 2-[1-(ethoxyimino) butyl]-5-[2-(ethylthio) propyl]-3-hydroxy-) as the selection medium. Whole-plant resistance was confirmed in greenhouse studies. This methodology allows the generation of non-transgenic plants resistant to herbicide. The plants obtained have not been released as cultivars. Mutation was employed on cultivars Argentine and Wilmington of *P. notatum* for improving turf quality, by using either seedlings or rhizomes with different mutagenic treatments: X-rays, gamma rays, ethyl methane sulphonate (Rios et al., 2013b) and by exposure of cells to sodium azide in tissue culture (Kannan et al., 2015). A total of 40 mutant plants were evaluated for utility turf, and some of them exhibited superior turf performance in comparison to the original cultivars (Rios et al., 2017).

## Cultivar Adoption

Approximately 94 cultivars have been released for the genus *Paspalum* L. (**Table S1**). Cultivars have been developed for different uses, e.g., cereal, turf or forage. In some cases, cultivars developed for forage are also used as utility turf, such as the cultivar Argentine of *P. notatum* Flüggé. Although the genus has nearly 310 species, only eight of these have produced cultivars. The species with most cultivars are *P. scrobiculatum* L., *P. vaginatum* Sw. and *P. notatum* (**Table S1**).

With 34 cultivars *P. scrobiculatum*, commonly known as ‘kodo millet’, is the species with the greatest number of cultivars. This is the only species of the genus that can be considered domesticated since it has been cultivated as an annual cereal in India for at least 3,000 years (de Wet et al., 1983). Currently, it is still grown as a major food source in India, particularly in the Deccan Plateau. It is also harvested as a secondary or wild cereal in India, Indonesia, Philippines, Thailand, Vietnam, and West Africa (Hariprasanna, 2017). It is also grown as a pasture crop in arid regions. Most cultivars were released as cereals in India during the past 30 years, and one, named Paltridge, was released for forage in Australia in 1966 (**Table S1**). *P. scrobiculatum* occurs in moist regions across the tropics and subtropics of the Old World (de Wet et al., 1983). It is a vigorous annual herb, 60 to 90 cm tall, which roots at lower nodes. The cultivated cytotype is tetraploid 2*n*=4*x*=40, and autogamous due to cleistogamy (Hariprasanna, 2017). Although several breeding techniques are being used in kodo millet, all released varieties are single plant selections from landraces or introduced germplasm from pure-line selection.

*Paspalum vaginatum,* known as ‘seashore paspalum’, is the *Paspalum* species with the second largest number of cultivars (**Table S1**). This species is cultivated as perennial turf in brackish and coastal environments across the tropics and subtropics around the world (Duncan and Carrow, 2000). The species has morphological characteristics that make it desirable as a turf, such as a spreading growth habit, tolerance to low mowing, deep green color, good density, and overall turf quality (Hanna et al., 2013). Its popularity as a warm-season turf mainly results from its salt tolerance, and ease of propagation (Duncan and Carrow, 2000; Eudy et al., 2017). Diploid (2*n* = 2*x* = 20), tetraploid (2*n* = 4*x* = 40) and hexaploid (2*n* = 6*x* = 60) cytotypes have been reported for *P. vaginatum*. However, the most widespread cytotype is the diploid, which is allogamous due to self-incompatibility (Hanna et al., 2013). Most cultivars released for *P. vaginatum* have been developed in USA, and the University of Georgia’s program has made an outstanding contribution. Germplasm collections from native environments or golf courses, evaluation in the target environment, selection and released of best clones were part of the breeding method used during the 21^st^ century. Nowadays, hybridization is commonly used in the most important breeding programs around the world (Raymer et al., 2007). Although, most cultivars are vegetatively propagated a few seed propagated cultivars are commercially available. Clonal cultivars are commercialized by sod farms around the world. In contrast, seed of seeded cultivars is produced by interplanting compatible parental clones and harvesting the F_1_ seed, as described by Hanna et al. (2013).

**Figure 4:**
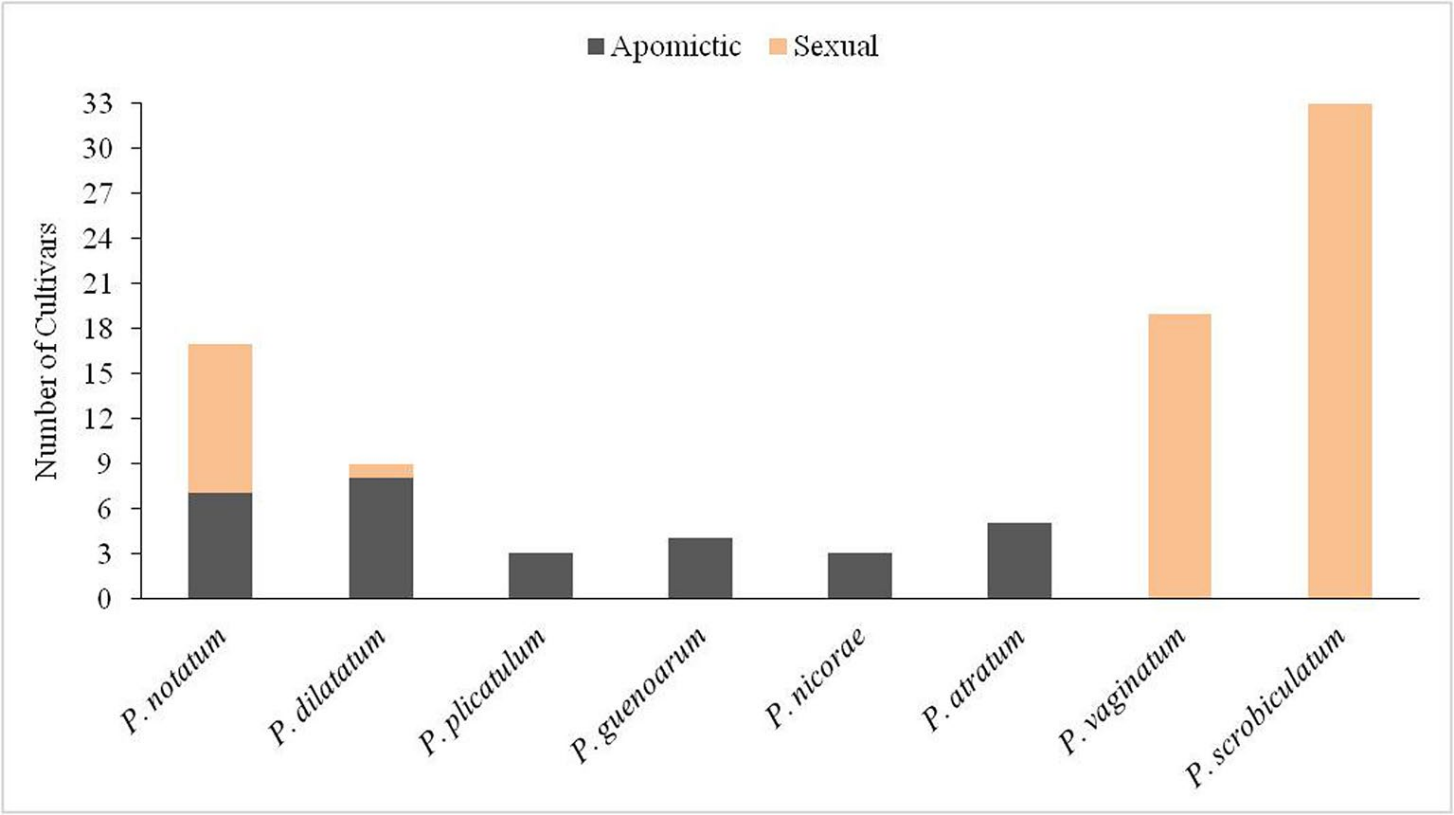
Number of cultivars in each *Paspalum* species. Apomictic cultivars are indicated with dark gray and sexual with orange.

*Paspalum notatum* is the third most important species of the genus in terms of the number of released cultivars (**Figure 4**). This species commonly known as bahiagrass is mainly cultivated as forage in the subtropical belt around the world, especially throughout Florida and the Coastal Plain and Gulf Coast Region of Southeastern USA (Blount and Acuña, 2009). Persistence under intense and frequent grazing, and adaptation to poor sandy soils are probably the reasons for its adoption as cultivated pasture (Gates et al., 2004). It is also sown or sodded extensively as utility turf, particularly in roadways, including interstate highways (Busey, 1989). Only cvv. Pensacola and Argentine are commercialized for utility turf, although they are mainly sold for forage. The species has mainly diploid (2*n* = 2*x* = 20) and tetraploid (2*n* = 4*x* = 40) types. The diploid is sexual and cross-pollinated; its natural distribution is restricted to northeastern Argentina (Burton, 1955; Burton, 1967). The tetraploid is an aposporous apomictic, and is naturally distributed from central Argentina to Northern Mexico (Gates et al., 2004). There are diploid and tetraploid cultivars, and breeding methods used to improve the two types differ. The widely known RRPS breeding method was created for improving diploid bahiagrass, and most diploid cultivars were developed using this method (Burton, 1989; Anderson et al., 2011). With the exception of cv. Boyero UNNE, all tetraploid cultivars are the result of direct selection from introduced germplasm in USA, Australia, and Japan (Blount and Acuña, 2009). Boyero UNNE is the first cultivar developed using hybridization to produce an apomictic F_1_ hybrid (Urbani et al., 2017).

*Paspalum dilatatum*, known as Dallisgrass, is the fourth species in terms of the number of cultivars (**Figure 4**). This species is used as forage with the particular aspect that it can be grown at higher latitudes than any other *Paspalum* species (Evers and Burson, 2004). It is currently grown in Australia, USA, and Uruguay. The species is native to the Americas and includes sexual tetraploid types, and apomictic pentaploid, and hexaploid types. Most cultivars released in USA, Argentina, Japan, and Australia are pentaploid (**Table S1**). There are also two hexaploid cultivars released in Uruguay and USA. All of these apomictic cultivars resulted from ecotype selection. There is one interesting case, which is the only tetraploid cultivar, cv. Primo, which resulted from interspecific hybridization between 4*x* sexual *P. dilatatum* and *P. urvillei* followed by several cycles of backcrossing to *P. dilatatum*. The objective was to transfer ergot tolerance from *P. urvillei* to *P. dilatatum* using this breeding technique (Schrauf et al., 2003).

The rest of the *Paspalum* spp. cultivars belong to the informal taxonomic group Plicatula. The group has 30 species; most of them are tropical or subtropical grasses with interesting qualities as forage (Zuloaga and Morrone, 2005). Rapid growth, high seed yield and aggressiveness for colonizing poor soils are characteristics that stand out for these species. Although there are conflicting taxonomic issues within the group, cultivars have been registered for four species, i.e., *P. atratum*, *P. guenoarum*, *P. plicatulum* and *P. nicorae* (**Table S1**). All cultivars within Plicatula are apomictic tetraploids and they were all released as forages.

*Paspalum atratum* is an upright grass with the ability to produce large forage yields concentrated during the warm season. It is adapted to a variety of soils from well drained sandy soils to poorly drained that stay saturated for several months (Kalmbacher et al., 1997; Hare et al., 1999a). The outstanding trait of *P. atratum* is its late flowering, which allows for an extended vegetative phase and a concentrated and uniform reproductive phase (Hare et al., 2001; Marcón et al., 2018). This results in pastures having high nutritive value during the growing season and high seed yield. It is possible that all released cultivars of *P. atratum* belong to the same accession even to the same genotype since they were all selected out of a germplasm collection from Campo Grande, Brazil. The species is cold sensitive, which limits its adoption in latitudes greater than 30° (Kalmbacher et al., 1997; Marcón et al., 2018). Currently it is cultivated in Northern Argentina and Indonesia (Hare et al., 1999a; Hare et al., 1999b; Altuve and Bendersky, 2003).

*Paspalum guenoarum* is also an erect species, which is also robust, like *P. atratum,* but is more cold-tolerant. It is adapted to well drained, acid soils (Ramírez, 1954). Currently, no seed is commercially available for any of the cultivars of *P. guenoarum*.

Seed of the three cultivars released in Australia for *P. plicatulum* was recently added to the germplasm bank of IBONE. Plants obtained from these seeds were grown in Corrientes (Argentina), and morphologically characterized. They were all classified as *P. lenticulare* Kunth instead of *P. plicatulum*. The main difference between these two species is the presence of ramifications in the inflorescence of *P. lenticulare* (Oliveira and Valls, 2008). *P. nicorae* is a rhizomatous grass, which exhibits an aggressive colonizing behavior when grown on sandy soils. No commercial seed is available for cultivars of *P. plicatulum* and *P. nicorae*.

Mode of reproduction has been a key trait for developing the 94 cultivars listed in **Table S1**. If the whole genus is considered, 68% of the released cultivars are sexual and 32% are apomictic. The lack of domestication stands out among *Paspalum* species with the exception of *P. scrobiculatum*. It is believed that apomixis is a barrier to domestication resulting in the absence of apomixis in most economically important crops (Darlington, 1939). Thus, it is not unexpected that *P. scrobiculatum* reproduces sexually since it has been cultivated as cereal for several thousand years. Sexuality in *P. scrobiculatum* has allowed the formation of a large number of self-pollinated and genetically uniform landraces in its area of cultivation, and that variation was used for selecting the released cultivars. In contrast, sexual reproduction in cross-pollinated *P. vaginatum* has been used for hybridization and generation of a large number of hybrids, which are evaluated as clonally propagated or, less commonly, seeded turf. Since *P. scrobiculatum* and *P. vaginatum* have the largest number of cultivars in the genus sexuality is common among *Paspalum* cultivars. Apomixis predominates in the natural distribution area of the rest of the species, i.e., *P. notatum*, *P. dilatatum*, *P. atratum*, *P. plicatulum*, *P. guenoarum*, and *P. nicorae* (Ortiz et al., 2013). The case of *P. notatum* is particular since although the diploid cytotype is restricted to a small area located in northern Argentina, it is well represented among cultivars (**Figure 4**). Adaptation of the diploid cytotype to the environment of Southeastern USA may be the key for its predominance and popularity. Tetraploid cultivars are also well represented for *P. notatum*, mainly through ecotypes and also with a new hybrid. The novel sexual tetraploid population recently created may allow for more efficient generation of new hybrids with a combination of desirable forage or turf characteristics (Zilli et al., 2018).

Apomixis has been the rule among cultivars of *P. dilatatum*, *P. atratum*, *P. plicatulum*, *P. guenoarum*, and *P. nicorae*. The recent generation of sexual tetraploid germplasm for the Plicatula group is allowing hybridization and gene flow among species, and may allow for the released of novel apomictic hybrid cultivars.

## Concluding Remarks

Polyploidy and apomixis are defining variables for allocation of plant diversity in *Paspalum*. Germplasm collection is expected to be more efficient if polyploid populations from contrasting environments are explored since the level of diversity depends on adaptation rather than geographical distances. Individual plant collections are suggested for most areas since monoclonal populations are common. Although of limited geographical distribution, rich levels of diversity are also present in sexual diploid populations and mixed diploid-tetraploid populations. In contrast to monoclonal populations, these mixed populations contained rich diversity and intensive plant collection is recommended for these particular areas.

There are several germplasm banks conserving *Paspalum* germplasm, but most species are conserved in Argentina, Brazil, and USA. There are also banks conserving a rich diversity for *P. scrobiculatum* in Asia due to its importance as a human food crop. Most of this germplasm is conserved as seed at low temperature and low humidity. The information for most banks is available online; however, this is not the case for a few of them. This review reports the complete list of accessions and species conserved in one of the most important banks for the genus, which is located in the region with the highest levels of diversity. Additionally, the recent generation of synthetic sexual tetraploid populations is expected to facilitate the conservation of entire species or taxonomic groups within single populations.

A variety of breeding methods are currently available for *Paspalum*. All of them are strongly dependent on mode of reproduction. Recent advances in breeding approaches developed for the apomictic species allow breeders to use the diversity locked in ecotypes for the generation of hybrids exhibiting heterosis for a variety of agronomically important traits. However, most current cultivars for apomictic species of *Paspalum* are the results of direct selection from accessions conserved and distributed among different regions. Moreover, novel breeding techniques developed for apomicts in *Paspalum* are expected to serve as a model for apomictic species of other genera and also for future apomictic crops if the trait is finally transferred to major crop species, such as maize and rice. The variable expressivity of apomixis is a key aspect to investigate in order to generate more efficient breeding methods.

Among the eight *Paspalum* species that are cultivated, *P. scrobiculatum* contains the greatest number of cultivars resulting from its relevance as a cereal in Asia. A species cultivated for turf, *P. vaginatum*, follows in importance since its turf quality and adaptation to the high salinity of coastal environments. *P. notatum* is the third in importance because of its multiple uses as forage, turf, and soil stabilization in acid and poor soils. The other five species have each been selected for forage production differing in their area of adaptation, e.g., *P. dilatatum* is adapted to mild temperate and sub-tropical environments while cultivars of the Plicatula group are largely adapted to tropical areas although some are adapted to sub-tropical areas. Sexual reproduction is the rule among the most economically important *Paspalum* cultivars belonging to *P. scrobiculatum*, *P. vaginatum*, and *P. notatum*. However, a large number of apomictic cultivars have been released mainly for forage. The adoption of an apomictic species or cultivar seems to be directly related to the stability over generations of agronomically important traits. The availability of novel breeding techniques for apomictic species is expected to have an impact mainly for future cultivars of *P. notatum* and species of the Plicatula group.

## Author Contributions

CA contributed designing the manuscript structure, writing, and compiling the manuscript. EM, AZ, EB, and FE contributed writing different sections of the manuscript. FM, MU, and CQ contributed organizing and summarizing the information on tables and figures.

## Conflict of Interest

The authors declare that the research was conducted in the absence of any commercial or financial relationships that could be construed as a potential conflict of interest.
