# Supplementary material for: Reproductive Systems in Paspalum: Relevance for Germplasm Collection and Conservation, Breeding Techniques, and Adoption of Released Cultivars
Source: Front Plant Sci. 2019 Nov 21;10:1377. doi: 10.3389/fpls.2019.01377 (PMC6881461; doi:10.3389/fpls.2019.01377)
Supplement: Supplementary file 1 [file Table_1.docx]

**Reproductive Systems in *Paspalum*: Relevance for Germplasm Collection and Conservation, Breeding Techniques, and Adoption of Released Cultivars**

*Carlos A. Acuña*, Eric J. Martínez, Alex L. Zilli, Elsa A. Brugnoli, Francisco Espinoza, Florencia Marcón, Mario H. Urbani, and Camilo L. Quarin*

*Instituto de Botánica del Nordeste, Consejo Nacional de Investigaciones Científicas y*

*Técnicas, Facultad de Ciencias Agrarias, Universidad Nacional del Nordeste, Corrientes, Argentina.*

***Correspondence:**

*Dr.* *Carlos A. Acuña*

*cacuna@agr.unne.edu.ar*

**TABLE S1.** List of released cultivars for the genus *Paspalum*.

| **Specie** | **Cultivar** | **Year of release** | **Release institution and country** | **Origin** | **Ploidy level and mode of reproduction** | **Use** | **Main attributes** | **Reference** |
| --- | --- | --- | --- | --- | --- | --- | --- | --- |
|  |  |  |  |  |  |  |  |  |
| *Paspalum notatum* | Pensacola | 1944 | Georgia SCS and Florida AES, USA | Argentina | 2n=2x=20, sexual | Forage, turf | Cold tolerant, long narrow leaves, early flowering. | Finlayson (1941) |
|  |  |  |  |  |  |  |  |  |
|  | Paraguay 22 | 1947 | USDA and Florida AES, USA | Paraguay | 2n=4x=40, apomictic | Forage | Resistant to ergot, more productive than cv. Paraguay. | McCloud (1953) |
|  |  |  |  |  |  |  |  |  |
|  | Argentine | 1950 | Florida AES, USA | Argentina | 2n=4x=40, apomictic | Forage, turf | Semi-erect, wide leaves, cold sensitive, ergot susceptible. | Killinger et al. (1951) |
|  |  |  |  |  |  |  |  |  |
|  | Tifhi 1 | 1958 | USDA-ARS and Georgia AES, USA | Hybrid | 2n=2x=20, sexual | Forage | F_1_ hybrid developed from Pensacola, higher yielding than Pensacola. | Hein (1958) |
|  |  |  |  |  |  |  |  |  |
|  | Tifhi 2 | 1958 | USDA-ARS and Georgia AES, USA | Hybrid | 2n=2x=20, sexual | Forage | F_1_ hybrid developed from Pensacola, higher yielding than Pensacola. | Evers and Burson (2004) |
|  |  |  |  |  |  |  |  |  |
|  | Nanpu | 1969 | Aichi Center, Japan | Japan | 2n=2x=20, sexual | Forage | Similar yield and palatability than Sinmoe, less productive than Nangoku, good winter survival. | Blount and Acuña (2009) |
|  |  |  |  |  |  |  |  |  |
|  | Wilmington | 1971 | SCS and Mississippi AES, USA | USA | 2n=4x=40, apomictic | Forage | Cold-hardy type, poor seed producer and less productive than Pensacola. | Blount and Acuña (2009) |
|  |  |  |  |  |  |  |  |  |
|  | Sinmoe | 1973 | Aichi Center, Japan |  | 2n=2x=20, sexual | Forage | Improved germination, good vigor, and earlier spring growth than Nanpu. | Blount and Acuña (2009) |
|  |  |  |  |  |  |  |  |  |
|  | Nangoku | 1983 | National Grassland RI, Japan | Japan | 2n=2x=20, sexual | Forage | Semi-erect growth habit, cold tolerant. | Takai and Komatsu (1998) |
|  |  |  |  |  |  |  |  |  |
|  | Competidor | 1986 | New South Wales DA, Australia | USA | 2n=4x=40, apomictic | Forage | Higher yielding, more palatable, fewer seed heads and more shade tolerant than Pensacola. | Wilson (1987) |
|  |  |  |  |  |  |  |  |  |
|  | Tifton 9 | 1987 | USDA-ARS, CPES, Georgia, USA | Hybrid | 2n=2x=20, sexual | Forage | Higher yielding, greater seedling vigor, and longer leaves than Pensacola. | Burton (1989) |
|  |  |  |  |  |  |  |  |  |
|  | Nan-ou | 1991 | Kagoshima Prefecture AS, Japan | USA | 2n=4x=40, apomictic | Forage | More palatable and greater forage yield in summer and early autumn than diploid cultivar Nangoku. | Takai and Komatsu (1998) |
|  |  |  |  |  |  |  |  |  |
|  | Riba | 1994 | New South Wales DA, Australia | Uruguay | 2n=4x=40, apomictic | Turf | Prostrate growth habit, dark-green leaf color and ergot resistant. | Loch and Ferguson (1999) |
|  |  |  |  |  |  |  |  |  |
|  | AU Sand Mountain | 1999 | Alabama AES, USA |  | 2n=2x=20, sexual | Forage | Cold tolerant, narrow leaves, fine tillers, and short inflorescence. | Blount and Acuña (2009) |
|  |  |  |  |  |  |  |  |  |
|  | UF-Riata | 2007 | UF-FAES, USA | Hybrid | 2n=2x=20, sexual | Forage | Low photoperiod sensitivity, cold tolerant, and increased forage production during the cool season. | Blount and Acuña (2009) |
|  |  |  |  |  |  |  |  |  |
|  | TifQuick | 2008 | UG and USDA-ARS, USA | Hybrid | 2n=2x=20, sexual | Forage | Rapid germination. | Anderson et al. (2011) |
|  |  |  |  |  |  |  |  |  |
|  | Boyero UNNE | 2012 | FCA-UNNE, Argentina | Hybrid | 2n=4x=40, apomictic | Forage | Semi erect growth habit, superior seasonal growth in comparison to cv. Argentine. | Urbani et al. (2017) |
|  |  |  |  |  |  |  |  |  |
| *P. dilatatum* | B-230 | 1951 | Louisiana AES, USA | USA | 2n=5x=50, apomictic | Forage | Better seed production than common dallisgrass. | Owen (1951) |
|  |  |  |  |  |  |  |  |  |
|  | B-430 | 1951 | Louisiana AES, USA | USA | 2n=5x=50, apomictic | Forage | Better seed production than common dallisgrass. | Owen (1951) |
|  |  |  |  |  |  |  |  |  |
|  | Grasslands Raki | 1979 | DSIR, New Zealand | Australia | 2n=5x=50, apomictic | Forage | Higher yielding than naturalized New Zealand ecotypes, poor seed production | Rumball (1983) |
|  |  |  |  |  |  |  |  |  |
|  | Natsugumo | 1980 | Kyushu National AES, Japan | Japan | 2n=5x=50, apomictic | Forage | Higher yielding than B230. | Yoshiyama et al. (1981) |
|  |  |  |  |  |  |  |  |  |
|  | La Estanzuela Chiru | Mid-1980s | CIA Alberto Boerger, Uruguay | Uruguay | 2n=6x=60, apomictic | Forage | More erect than common dallisgrass and produce more forage. | Evers and Burson (2004) |
|  |  |  |  |  |  |  |  |  |
|  | Magnifi 217 Pampeano INTA | 1988 | INTA-Castelar, Argentina |  | 2n=5x=50, apomictic | Forage | Slow establishment, poor seed production and susceptible to ergot. | INASE (1988) |
|  |  |  |  |  |  |  |  |  |
|  | Relincho | 2003 | UBA-FA, Argentina |  | 2n=5x=50, apomictic | Forage | Fast establishment, waterlogging tolerant, poor seed production and susceptible to ergot. | INASE (2003) |
|  |  |  |  |  |  |  |  |  |
|  | Sabine | 2008 | USDA-ARS, Louisiana SUAC, and Texas AgriLife Research, USA |  | 2n=6x=60, apomictic | Forage | Produced more forage and is more persistent under defoliation than common dallisgrass. | Burson et al. (2009) |
|  |  |  |  |  |  |  |  |  |
|  | Primo-FAUBA | 2013 | FAUBA, Argentina | Hybrid | 2n=4x=40, sexual | Forage | Ergot resistant, cold tolerant, higher yield than Relincho. | INASE (2013) |
|  |  |  |  |  |  |  |  |  |
| *P. plicatulum* | Rodd´s Bay | 1963 | CSIRO, Australia | Guatemala | 2n=4x=40, apomictic | Forage | Erect growth habit, leaves more narrow than other two cultivars, hairs on leaf-blades, cold susceptible, highly tolerant to waterlogging for short periods. | Oram (1990) |
|  |  |  |  |  |  |  |  |  |
|  | Hartley | 1963 | CSIRO, Australia | Brazil | 2n=4x=40, apomictic | Forage | Broader leaves, leaf-blades glabrous, higher nutritive value and less seed production than Rodd´s Bay. Also released in Zimbabwe as Beehive *Paspalum*. | Oram (1990), Loch and Ferguson (1999) |
|  |  |  |  |  |  |  |  |  |
|  | Bryan | 1975 | CSIRO, Australia | Puerto Rico | 2n=4x=40, apomictic | Forage | Broader leaves and less upright growth habit than Rodd's Bay, hairs on leaf blades, more drought tolerant than other two cultivars. | Oram (1990) |
|  |  |  |  |  |  |  |  |  |
| *P. guenoarum* | Pasto Rojas | 1953 | IAN, Paraguay | Paraguay | 2n=4x=40, apomictic | Forage | Easy establishment, adapted to a wide range of soils, tolerant to animal trampling, cold and drought tolerant. | Ramírez (1954) |
|  |  |  |  |  |  |  |  |  |
|  | Wintergreen | 1963 | Zimbabwe |  | 2n=4x=40, apomictic | Forage | Lost favor because difficult to establish in dry years. | Loch and Ferguson (1999) |
|  |  |  |  |  |  |  |  |  |
|  | Pasto Ramírez | 1983 | INTA, Argentina | Argentina | 2n=4x=40, apomictic | Forage | More tolerant to cold than Pasto Rojas and not adapted to flooding. | Pérego (2010) |
|  |  |  |  |  |  |  |  |  |
|  | Chané-FCA | 2002 | FCA-UNNE, Argentina | Bolivia | 2n=4x=40, apomictic | Forage | Only flowers during the fall, cold sensitive, good forage quality and seed production. | INASE (2002) |
|  |  |  |  |  |  |  |  |  |
| *P. nicorae* | Doncorae | 1993 | USDA-ARS, USA | Brazil | 2n=4x=40, apomictic | Cover crop | Rapid seedling establishment, vigorous growth habit, and winter hardiness. | Belt and Englert (1999), Anderson and Sharp (1994) |
|  |  |  |  |  |  |  |  |  |
|  | Blue Eve | 1999 | Enviroseeds, Australia | Argentina | 2n=4x=40, apomictic | Turf | Plant mutant from Blue Dawn, turf-type with a finer, lower-growing sward with fewer seed heads. | Evers and Burson (2004), DPI (2007) |
|  |  |  |  |  |  |  |  |  |
|  | Blue Dawn | 2001 | Progressive Seeds, Australia | Argentina | 2n=4x=40, apomictic | Turf, forage | Dual purpose pasture, erect growth, long, deep and vigorous rhizomes, cold and drought tolerant. | Evers and Burson (2004), DPI (2007) |
|  |  |  |  |  |  |  |  |  |
| *P. atratum* | Suerte | 1995 | Florida AES, USA | Brazil | 2n=4x=40, apomictic | Forage | Produces good quality forage throughout growing season, produces good quality seed in fall, tolerates flooding. | Kalmbacher et al. (1997) |
|  |  |  |  |  |  |  |  |  |
|  | Hi-Gane | 1996 | Australia | Brazil | 2n=4x=40, apomictic | Forage | Actually is Suerte but is marketed as Hi-Gane in Australia. | Adapted from Evers and Burson (2004) |
|  |  |  |  |  |  |  |  |  |
|  | Cambá-FCA | 1997 | FCA-UNNE, Argentina | Brazil | 2n=4x=40, apomictic | Forage | Selected from Brazilian germplasm (BRA 009610), produces good quality forage throughout growing season, produces good quality seed in fall, tolerates flooding. | INASE (1997) |
|  |  |  |  |  |  |  |  |  |
|  | Ubon | 1998 | Ubon Ratchathani University, Thailand | Brazil | 2n=4x=40, apomictic | Forage | Selected from Brazilian germplasm (BRA 009610), grown on abandoned rice fields, tolerates flooding, used primarily for dairy animals, good seed production. | Adapted from Evers and Burson (2004) |
|  |  |  |  |  |  |  |  |  |
|  | Pojuca | 2000 | EMBRAPA, Brazil | Brazil | 2n=4x=40, apomictic | Forage | Selected from Brazilian germplasm (BRA 009610), same characteristics as for Cambá above. | Adapted from Evers and Burson (2004) |
|  |  |  |  |  |  |  |  |  |
| *P. vaginatum* | Saltene | 1951 | The Turf Farm, Waneroo Turf Farm, Australia | South Africa | 2n=2x=20, sexual | Turf | Lawn-type, intermediate texture. | Duncan and Carrow (2000) |
|  |  |  |  |  |  |  |  |  |
|  | Salpas | 1951 |  | Australia | 2n=2x=20, sexual | Turf | Intermediate leaf texture. | Duncan and Carrow (2000) |
|  |  |  |  |  |  |  |  |  |
|  | Futurf | 1972 |  | Australia | 2n=2x=20, sexual | Turf | Intermediate leaf texture. | Duncan and Carrow (2000) |
|  |  |  |  |  |  |  |  |  |
|  | Adalayd | 1975 | UG, USA | Australia | 2n=2x=20, sexual | Turf | Intermediate leaf texture. | Duncan and Carrow (2000) |
|  |  |  |  |  |  |  |  |  |
|  | Fidalayel | 1975 |  | USA | 2n=2x=20, sexual | Turf | Intermediate leaf texture. | Duncan and Carrow (2000) |
|  |  |  |  |  |  |  |  |  |
|  | SeaDwarf | 1980s | Environmental Turf Inc., USA | USA | 2n=2x=20, sexual | Turf | Fine-textured, bright green leaves with high shoot density. | Brosnan and Deputy (2008) |
|  |  |  |  |  |  |  |  |  |
|  | Tropic Shore | 1991 | USDA and University of Hawaii, USA | USA | 2n=2x=20, sexual | Turf | Coarse leaf texture. | Duncan and Carrow (2000), USDA-NRCS (2013) |
|  |  |  |  |  |  |  |  |  |
|  | Salam | 1998 | Southern Turf Nurseries, USA | USA | 2n=2x=20, sexual | Turf | Fine-textured, dark green leaves with high shoot density, susceptible to dollar spot. | Duncan and Carrow (2000), Brosnan and Deputy (2008) |
|  |  |  |  |  |  |  |  |  |
|  | See Isle 1 | 1999 | UG, USA | Argentina | 2n=2x=20, sexual | Turf | Fine-textured, dark green leaves with high shoot density, drought tolerant. | Duncan (2002), Brosnan and Deputy (2008) |
|  |  |  |  |  |  |  |  |  |
|  | See Isle 2000 | 1999 | UG, USA | USA | 2n=2x=20, sexual | Turf | Fine-textured, dark green leaves with high shoot density, susceptible to dollar spot. | Duncan (2002), Brosnan and Deputy (2008) |
|  |  |  |  |  |  |  |  |  |
|  | Brazoria | 1999 | USDA-Natural Resources CS, USA | USA | 2n=2x=20, sexual | Turf | Semi-aquatic, rapid-growing, decumbent grass. | USDA-NRCS (2015) |
|  |  |  |  |  |  |  |  |  |
|  | Durban CC | 2000 | UG, USA | South Africa | 2n=2x=20, sexual | Turf | Fine-textured. | Duncan and Carrow (2000) |
|  |  |  |  |  |  |  |  |  |
|  | SeaWay | 2002 | Environmental Turf Inc., USA | USA | 2n=2x=20, sexual | Turf | Fine-textured, superior tolerance to salt, drought, and soil hypoxic conditions. | Bennett and DePew (2002a) |
|  |  |  |  |  |  |  |  |  |
|  | SeaGreen | 2002 | Environmental Turf Inc., USA | USA | 2n=2x=20, sexual | Turf | Fine-textured, superior tolerance to salt, drought, and soil hypoxic conditions. | Bennett and DePew (2002b) |
|  |  |  |  |  |  |  |  |  |
|  | SeaSpray | 2005 | Pure Seed Testing Inc. and UG, USA | USA | 2n=2x=20, sexual | Turf | The only cultivar that can be established from seed.  Medium-textured, bright green leaves with high shoot density, less susceptible to dollar spot than other seashore cultivars. | Brosnan and Deputy (2008) |
|  |  |  |  |  |  |  |  |  |
|  | Aloha | 2005 | Florida and Hawaii AES, USA | USA | 2n=2x=20, sexual | Turf | Fast rate of establishment and ground coverage, dark and deep green leaf color, and superior resistance to the greenbug aphid. | Scully et al. (2011) |
|  |  |  |  |  |  |  |  |  |
|  | Sea Isle Supreme | 2005 | UG, USA | USA | 2n=2x=20, sexual | Turf | Low-growing and rapidly spreading semi dwarf type, excellent salt tolerance. | Raymer et al. (2007) |
|  |  |  |  |  |  |  |  |  |
|  | Platinum TE | 2008 | Turf Ecosystems, LLC, USA | USA | 2n=2x=20, sexual | Turf | Semi-dwarf growth habit, rapid establishment, good resistance to foliar diseases. | Duncan (2008) |
|  |  |  |  |  |  |  |  |  |
|  | SeaStar | 2015 | UG, USA | USA | 2n=2x=20,  sexual | Turf | Excellent green turf color, non-dwarf, rapidly spreading growth habit, good salt tolerance. | Raymer et al. (2015) |
| *P. scrobiculatum* | Paltridge | 1966 | Queensland DPI, Australia | Zimbabwe | 2n=2x=40, sexual | Forage | Slightly stoloniferous, medium to low yield, very palatable and highly digestible, low crude protein content. | Oram (1990) |
|  |  |  |  |  |  |  |  |  |
|  | PLR1 | 1942 | TNAU, India | India | 2n=2x=40, sexual | Cereal | Long duration, low yielding. | ICAR (2014) |
|  | CO 1 | 1953 | TNAU, India | India | 2n=2x=40, sexual | Cereal | Profuse tillering, long duration. | ICAR (2014) |
|  | CO 2 | 1970 | TNAU, India | India | 2n=2x=40, sexual | Cereal | Profuse tillering, long duration, low yielding. | ICAR (2014) |
|  | Niwas 1 | 1971 | JNKVV, India | India | 2n=2x=40, sexual | Cereal | Erect plant type, early duration. | ICAR (2014) |
|  | JKN 101 | 1976 | JNKVV, India | India | 2n=2x=40, sexual | Cereal | Erect plant type, early duration. | ICAR (2014) |
|  | GK 1 | 1977 | GAU, India | India | 2n=2x=40, sexual | Cereal | High yielding. | ICAR (2014) |
|  | JNK 364 | 1977 | JNKVV, India | India | 2n=2x=40, sexual | Cereal | Early duration, tolerant to drought. | ICAR (2014) |
|  | CO 3 | 1980 | TNAU, India | India | 2n=2x=40, sexual | Cereal | Semi-erect plants. | ICAR (2014) |
|  | K 1 | 1982 | ARS-Kovilpatti, India | India | 2n=2x=40, sexual | Cereal | Profuse tillering. | ICAR (2014) |
|  | JK 1 | 1982 | JNKVV, India | India | 2n=2x=40, sexual | Cereal | Profuse tillering, high yielding. | ICAR (2014) |
|  | JK 2 | 1982 | JNKVV, India | India | 2n=2x=40, sexual | Cereal | Profuse tillering, high yielding. | ICAR (2014) |
|  | PSC 1 | 1986 | PCC, India | India | 2n=2x=40, sexual | Cereal | Dense spikelets, high yielding, non-lodging, moderately tolerant to shootfly and drought. | ICAR (2014) |
|  | JK 41 | 1986 | JNKVV, India | India | 2n=2x=40, sexual | Cereal | Erect plant type, resistant to head smut and moderately drought tolerant, suitable for inter/mixed cropping. | ICAR (2014) |
|  | JK 76 | 1989 | JNKVV, India | India | 2n=2x=40, sexual | Cereal | Earliness, moderately tolerant to shootfly and tolerant to drought. | Hariprasanna (2017) |
|  |  |  |  |  |  |  |  |  |
|  | JK 62 | 1989 | JNKVV, India | India | 2n=2x=40, sexual | Cereal | Earliness, high yield, resistant to head smut and bacterial blight. | Hariprasanna (2017) |
|  |  |  |  |  |  |  |  |  |
|  | GPUK 3 | 1991 | UAS, India | India | 2n=2x=40, sexual | Cereal | High yielding, earliness, resistant to head smut and moderately tolerant to low moisture stress. | Hariprasanna (2017) |
|  |  |  |  |  |  |  |  |  |
|  | APK 1 | 1993 | ARS, India | India | 2n=2x=40, sexual | Cereal | High seed yield, resistant to ergot, smut and tolerant to stem borer. | Hariprasanna (2017) |
|  |  |  |  |  |  |  |  |  |
|  | GK 2 | 1993 | GAU, India | India | 2n=2x=40, sexual | Cereal | Tolerant to drought. | Hariprasanna (2017) |
|  |  |  |  |  |  |  |  |  |
|  | KMV 20 | 1996 | TNAU, India | India | 2n=2x=40, sexual | Cereal | Tolerant to sheath blight and to drought. | Hariprasanna (2017) |
|  |  |  |  |  |  |  |  |  |
|  | KK 1 | 1999 | CSAUA&T, India | India | 2n=2x=40, sexual | Cereal | Synchronous tillering, non-lodging, resistant to head smut, tolerant to drought and salinity. | ICAR (2014) |
|  | JK 155 | 2000 | JNKVV, India | India | 2n=2x=40, sexual | Cereal | Resistant to head smut and shoot fly. | Hariprasanna (2017) |
|  |  |  |  |  |  |  |  |  |
|  | JK 48 | 2001 | JNKVV, India | India | 2n=2x=40, sexual | Cereal | Tolerance to head smut and high yielding. | Hariprasanna (2017) |
|  |  |  |  |  |  |  |  |  |
|  | KK 2 | 2002 | Chandra Shekhar Azad UAT, India | India | 2n=2x=40, sexual | Cereal | Resistant to drought and lodging, suitable for saline condition. | Hariprasanna (2017) |
|  |  |  |  |  |  |  |  |  |
|  | JK 439 | 2002 | JNKVV, India | India | 2n=2x=40, sexual | Cereal | High yielding, moderately resistant to smut and shoot fly, suitable for shallow soil with marginal fertility. | Hariprasanna (2017) |
|  |  |  |  |  |  |  |  |  |
|  | JK 13 | 2006 | JNKVV, India | India | 2n=2x=40, sexual | Cereal | Moderately resistant to head smut, resistant to shoot fly. | Hariprasanna (2017) |
|  |  |  |  |  |  |  |  |  |
|  | JK 65 | 2008 | JNKVV, India | India | 2n=2x=40, sexual | Cereal | High yielding, resistant to head smut. | Hariprasanna (2017) |
|  |  |  |  |  |  |  |  |  |
|  | JK 106 | 2008 | JNKVV, India | India | 2n=2x=40, sexual | Cereal | Resistant to head smut and shoot fly. | Hariprasanna (2017) |
|  |  |  |  |  |  |  |  |  |
|  | JK 36 | 2009 | JNKVV, India | India | 2n=2x=40, sexual | Cereal | Very early maturation, low yielding, moderately tolerant to shoot fly. | Hariprasanna (2017) |
|  |  |  |  |  |  |  |  |  |
|  | JK 98 | 2010 | JNKVV, India | India | 2n=2x=40, sexual | Cereal | Early duration, high yielding, moderately resistant to head smut, tolerant to shoot fly. | Hariprasanna (2017) |
|  |  |  |  |  |  |  |  |  |
|  | DPS 9‐1 | 2011 | JNKVV, India | India | 2n=2x=40, sexual | Cereal | High yielding, resistant to head smut. | Hariprasanna (2017) |
|  |  |  |  |  |  |  |  |  |
|  | Indira kodo 1 | 2012 | IGKV, India | India | 2n=2x=40, sexual | Cereal | Moderately susceptible to head smut, highly responsive to fertilizers, suitable for late sown condition. | Hariprasanna (2017) |
|  |  |  |  |  |  |  |  |  |
|  | TNAU 86 | 2012 | TNAU, India | India | 2n=2x=40, sexual | Cereal | Early maturation, non-lodging, high yielding and milling. | Hariprasanna (2017) |
|  |  |  |  |  |  |  |  |  |
|  | RK 390‐25 | 2012 | JNKVV, India | India | 2n=2x=40, sexual | Cereal | High yielding, moderately resistant to head smut. | Hariprasanna (2017) |
|  |  |  |  |  |  |  |  |  |

**REFERENCES**

Anderson, J., and Sharp, W.C. (1994). Grass varieties in the United States. Washington: U.S. Department of Agriculture.

Anderson, W.F., Gates, R.N., and Hanna, W.W. (2011). Registration of ‘TifQuik’ Bahiagrass. J. Plant Regist. 5, 147-150. doi: 10.3198/jpr2010.07.0427crc

Belt, S.V., and Englert, J.M. (1999). Improved conservation plant materials released by NRCS and cooperators through December 2007. 2008 USDA-NRCS-NPMC, Beltsville, Maryland, U.S.A. Online: <http://www.nrcs.usda.gov/Internet/FSE_DOCUMENTS/nrcs144p2_064674.pdf>

Bennett, S.T., and DePew, M. (2002a). Seashore Paspalum “SFX-14”. U.S Patent No 13,105 P2. Washington, DC: U.S. Patent and Trademark Office.

Bennett, S.T., and DePew, M. (2002b). Seashore Paspalum “SGX-6”. U.S Patent No 13,100 P2. Washington, DC: U.S. Patent and Trademark Office.

Blount, A.R., and Acuña, C.A. (2009). Bahiagrass, in Genetic resources, chromosome engineering, and crop improvement series: Forage crops, ed. R.J. Singh (Boca Raton, FL: CRC Press), 5, 81-101.

Brosnan, J.T., and Deputy, J. (2008). Seashore Paspalum. Cooperative Extension Service, College of Tropical Agriculture and Human Resources, University of Hawaii, Honolulu, Hawaii.

Burson, B.L., Venuto, B.C., and Hussey, M.A. (2009). Registration of ‘Sabine’ dallisgrass. J. Plant Regist. 3, 132-137. doi: 10.3198/jpr2008.11.0648crc

Burton, G.W. (1989). Registration of Tifton 9 Pensacola bahiagrass. Crop Sci. 29, 1326.

Department of Primary Industries (DPI). (2007). Pasture Varieties used in NSW 2006-2007. New South Wales: NSW Department of Primary Industries.

Duncan, R.R., and Carrow, R.N. (2000). Seashore *Paspalum*: The environmental turfgrass. John Wiley & Sons, Hoboken, NJ.

Duncan, R.R. (2002). Seashore Paspalum plant “Sea Isle 2000”, U.S. Patent No 12,625 P2. Washington, DC: U.S. Patent and Trademark Office

Duncan, R.R. (2008). Seashore Paspalum plant named “TE-13”. U.S Patent No 19,224 P3. Washington, DC: U.S. Patent and Trademark Office

Evers, G.W. and Burson, B.L. (2004). Dallisgrass and other *Paspalum* species, in Warm-season (C4) grasses, eds. L.E. Moser, B.L. Burson, L.E. Sollenberger (Madison, Wisconsin, USA: ASA, CSSA, SSSA Press), 45: 681-713.

Finlayson, E.H. (1941). Pensacola-A new fine leaved bahia. Southern Seedsman. 4, 12-9.

Hariprasanna, K. (2017). Kodo Millet, *Paspalum scrobiculatum* L, in Millets and Sorghum: Biology and Genetic Improvement, ed J.V. Patil (Hyderabad, India: John Wiley & Sons Ltd. Press),199-225.

Hein, M.A. (1958). Registration of varieties and strains of grasses. Agron. J. 50, 399-401.

Indian Council of Agricultural Research (ICAR). (2014). Compendium of released varieties in small millets. GKVK, Bangalore, India. pp. 103-134. <https://www.dhan.org/smallmillets/docs/report/Compendium_of_Released_Varieties_in_Small_millets.pdf>

Instituto Nacional de Semillas (INASE). (1988). Magnifi 217 Pampero INTA. Registro Nacional de Cultivares N° 1886. Buenos Aires, Argentina. <https://gestion.inase.gov.ar/consultaGestion/gestiones>

Instituto Nacional de Semillas (INASE). (1997). Cambá FCA. Registro Nacional de Cultivares N° 4655. Buenos Aires, Argentina. <https://gestion.inase.gov.ar/consultaGestion/gestiones>

Instituto Nacional de Semillas (INASE). (2002). Chané FCA. Registro Nacional de Cultivares N° 7545. Buenos Aires, Argentina. <https://gestion.inase.gov.ar/consultaGestion/gestiones>

Instituto Nacional de Semillas (INASE). (2003). Relincho. Registro Nacional de Cultivares N° 7774. Buenos Aires, Argentina. <https://gestion.inase.gov.ar/consultaGestion/gestiones>

Instituto Nacional de Semillas (INASE). (2013). Primo-FAUBA. Registro Nacional de Cultivares N° 12252. Buenos Aires, Argentina. <https://gestion.inase.gov.ar/consultaGestion/gestiones>

Kalmbacher, R.S., Brown, W.F., Colvin, D.L., Dunavin, L.S., Kretschmer, A.E., Jr., Martin, F.G., Mullahey, J.J., and Rechcigl, J.E. (1997). 'Suerte' atra paspalum: Its management and utilization. Circular S-397, Florida Agriculture Experimental Station, Gainesville.

Killinger, G.B., Ritchey, G.E., Blickensderfer, C.B., and Jackson, W. (1951). Argentine bahiagrass. Agricultural Experiment Station Annual Report. University of Florida, Gainesville.

Loch, D.S., and Ferguson, J.E. (1999). Tropical and Subtropical forage seed production: An overview, in Forage seed production. Vol. 2: Tropical and subtropical species, eds. D.S. Loch and J.E. Ferguson (Wallingford, Oxon, UK CABI Press), 1-40.

McCloud, D.E. (1953). Forage and cover plant introduction by the Florida Agricultural Experiment Station. Soil Crop Science Society of Florida Proceedings. 13, 32-38.

Oram, R.N. (1990). Register of Australian herbage plant cultivars. Melbourne: CSIRO Publications.

Owen, C.R. (1951). Improvement of native dallisgrass in Louisiana. Louisiana Agriculture Experimental Station, Baton Rouge. Bulletin 449.

Pérego, J.L. (2010). Pasto Ramírez. Viejas forrajeras para nuevas pasturas. INTA N° 457. Buenos Aires, Argentina: editorial INTA.

Ramírez, J.R. (1954). EI Pasto Rojas: Una gramínea forrajera promisoria en el Paraguay. Revista Argentina de Agronomía. 21, 84-101.

Raymer, P.L., Braman, S.K., Burpee, L.L., Carrow, R.N., Chen, Z., and Murphy, T.R. (2007). Seashore Paspalum: breeding a turfgrass for the future. USGA Turfgrass and Environmental Research Online. 6 (21), 1-8.

Raymer, P.L, Burpee, L.L, Carrow, R.N, and Schwartz, B.M. (2015). U.S. Patent No 25,761 P3. Washington, DC: U.S. Patent and Trademark Office.

Rumball, R. (1983). Other grasses, in Plant breeding in New Zealand, eds. G.S. Wratt and H.C. Smith (Wellington, NZ: Butterworths NZ Press), 263-270.

Scully, B.T., Nagata, R.T., Sistrunk, D.M., Cherry, R.H., Nuessly, G.S., Kenworthy, K.E., and DeFrank, J. (2011). Registration of ‘Aloha’ Seashore Paspalum. J. Plant Regist. 5, 22-26. doi: 10.3198/jpr2009.09.0498crc

Takai, T., and Komatsu, T. (1998). Comparison on physical strength and structure of the leaf blade between Nan-ou and Nangoku varieties of Bahiagrass (*Paspalum notatum* Flüggé). Sochi Shikenjo Kenkyu Hokoku 56, 13-20. {a} Hokkaido Natl. Agric. Exp. Stn., Hitsujigaoka, Toyohira-ku, Sapporo, Hokkaido 062-8555, Japan.

Urbani, M.H., Acuña, C.A., Doval, D.W., Sartor, M.E., Galdeano, F., Blount, A.R., Quesenberry, K.H., Mackowiak, C.L., and Quarin, C.L. (2017). Registration of ‘Boyero UNNE’ Bahiagrass. J. Plant Regist. 11, 26-32. doi: 10.3198/jpr2016.04.0021crc

USDA-Natural Resources Conservation Service. (2013). Release Brochure for release ‘Tropic Shore’ Seashore Paspalum (*Paspalum vaginatum*). Hoolehua PMC: Holehua, Hawaii 96729.

USDA-Natural Resources Conservation Service. (2015), Release Brochure for Brazoria Seashore Paspalum, (*Paspalum vaginatum* Sw.). Golden Meadow Plant Materials Center, Galliano, LA 70354.

Wilson, G.P.M. (1987). *Paspalum notatum* Flüggé (Bahia grass). cv. Competidor (Reg. no. A-7c-1). Trop. Grassl. 21, 93-94.

Yoshiyama, T., Tsurumi, Y., Nakashima, K., Matsumoto, S., Terada, Y., Aoi, K., and Hirai, T. (1981). A new dallisgrass cuItivar 'Natsugumo'. Bulletin Kyushu National Agriculture Experimental Station. 21, 353-369.
